# Supplementary material for: A systematic review of barriers and enablers to South Asian women’s attendance for asymptomatic screening of breast and cervical cancers in emigrant countries
Source: BMJ Open. 2018 Jul 7;8(7):e020892. doi: 10.1136/bmjopen-2017-020892 (PMC6042536; doi:10.1136/bmjopen-2017-020892)
Supplement: Author's manuscript [file bmjopen-2017-020892.draft_revisions.pdf]

BMJ Open is committed to open peer review. As part of this commitment we make the peer review history of every article we publish publicly available.

When an article is published we post the peer reviewers' comments and the authors' responses online. We also post the versions of the paper that were used during peer review. These are the versions that the peer review comments apply to.

The versions of the paper that follow are the versions that were submitted during the peer review process. They are not the versions of record or the final published versions. They should not be cited or distributed as the published version of this manuscript.

BMJ Open is an open access journal and the full, final, typeset and author-corrected version of record of the manuscript is available on our site with no access controls, subscription charges or pay-per-view fees (http://bmjopen.bmj.com).

If you have any questions on BMJ Open's open peer review process please email info.bmjopen@bmj.com

BMJ Open

A systematic review of barriers and enablers to South Asian women's attendance for asymptomatic screening of breast and cervical cancers in emigrant countries

Journal:	*BMJ Open*
Manuscript ID	bmjopen-2017-020892
Article Type:	Research
Date Submitted by the Author:	01-Dec-2017
Complete List of Authors:	Saini, Pooja; University of Liverpool, Health Services Research<br>Anderson de Cuevas, Rachel; University of Liverpool, Public Health & Policy<br>Roberts, Deborah; Royal Liverpool and Broadgreen University Hospitals NHS Trust<br>Beaver, Kinta; University of Central Lancashire; University of Central Lancashire<br>Chandrashekar, Mysore; Royal Liverpool and Broadgreen University Hospitals NHS Trust<br>Jain, Anil; University Hospital of South Manchester NHS Foundation Trust<br>Kotas, Eleanor; University of Liverpool, Health Services Research<br>Tahir, Naheed; University of Liverpool , Health Services Research<br>Ahmed, Saiqa; University of Liverpool, Health Services Research<br>Brown, Stephen; University of Liverpool, Psychological Sciences
**Primary Subject Heading**:	Oncology
Secondary Subject Heading:	Research methods, Public health, Health services research, Evidence based practice
Keywords:	South Asian women, asymptomatic screening, breast cancer, cervical cancer, mammography, smear

SCHOLARONE™
Manuscripts

A systematic review of barriers and enablers to South Asian women's attendance for asymptomatic screening of breast and cervical cancers in emigrant countries

Correspondence: Pooja Saini, Department of Health Services Research, 1st Floor, Waterhouse Building, Brownlow Hill, University of Liverpool, L69 3GS. Tel: 01517949966. Email: psaini@liverpool.ac.uk

Pooja Saini*, NIHR Collaboration for Leadership in Applied Health Research and Care North West Coast, University of Liverpool, Waterhouse Building, Brownlow Hill, University of Liverpool, L69 3GS, UK

Rachel Anderson de Cuevas*, University of Liverpool, Whelan Building, Brownlow Hill, University of Liverpool, L69 3GS, UK

*Joint first authorship. Pooja Saini and Rachel Anderson de Cuevas contributed equally.

Deborah Roberts, Royal Liverpool and Broadgreen Hospital NHS Trust, Prescot St, Liverpool, L7 8XP, UK

Kinta Beaver, University of Central Lancashire, School of Health Sciences, Brook Building, Preston, PR1 2HE, UK

Mysore Chandrashekar, Royal Liverpool and Broadgreen Hospital NHS Trust, Prescot St, Liverpool, L7 8XP, UK

Anil Jain, The Nightingale Centre and Genesis Prevention centre, University Hospital of South Manchester NHS Foundation Trust, Southmoor Rd, Wythenshawe, Manchester, M23 9QZ, UK

Eleanor Kotas, University of Liverpool, Whelan Building, Brownlow Hill, University of Liverpool, L69 3GS, UK

Naheed Tahir, NIHR Collaboration for Leadership in Applied Health Research and Care North West Coast Public Advisor, University of Liverpool, Liverpool, UK

Saiqa Ahmed, NIHR Collaboration for Leadership in Applied Health Research and Care North West Coast Public Advisor, Liverpool, UK

Stephen L. Brown, University of Liverpool, Whelan Building, Brownlow Hill, University of Liverpool, L69 3GS, UK

Word count: 4668 words

For peer review only - http://bmjopen.bmj.com/site/about/guidelines.xhtml

Abstract

Objectives: The aim of this review was to identify the cultural, social, structural and behavioural factors that influence asymptomatic breast and cervical cancer screening rates in South Asian populations in order to improve screening rates and propose priorities for further research.

Design: A computerised A systematic review of the literature for inductive, comparative, prospective and intervention studies. We searched the following databases: MEDLINE/In-Process; Web of Science; EMBASE; SCOPUS; CENTRAL; CDSR; CINAHL; PsycINFO and PsycARTICLES. Studies were included for attendance at asymptomatic screening of female breast and/or cervical cancers by routine mammography and cervical smear testing for South Asian women and those published in the English language. The Framework Analytic method was used and themes were drawn out following the Thematic Analysis method.

Settings: Asymptomatic breast or cervical screening

Participants: South Asian women, including Bangladeshi, Indian, Pakistani, Sri Lankan, Bhutanese, Maldivian and Nepali populations.

Results: 44 included studies were published between 1991 and 2016. Sample sizes ranged from 25 to 38,733 and participants had a mean age of 18 to 83 years. Our review showed that South Asian women generally had lower screening rates than host country women. South Asian women had poorer knowledge of cancer and cancer prevention and experienced more barriers to screening. Cultural practices and assumptions influenced understandings of cancer and prevention, emphasising the importance of host country cultures and healthcare systems.

Conclusions: High quality research on screening attendance is required using prospective designs, where objectively-validated attendance is predicted from cultural understandings,

For peer review only - http://bmjopen.bmj.com/site/about/guidelines.xhtml

beliefs, norms and practices; thus informing policy on targeting relevant public health messages to the south Asian communities about screening for cancer.

Systematic Review Registration: PROSPERO CSD 42015025284

Strengths and limitations of this study

- Separate outcomes were compared of integrative reviews of inductive, predictive, comparative and intervention studies to assess consistencies between methods.
- Deductive studies were either atheoretical or used generic health psychology theories, such as the health belief model, that were validated on western samples and not adapted for South Asian populations.
- Studies were primarily conducted in Canada and the USA where a payment-oriented healthcare is available which may not be comparable to other health services.
- Due to the small number of published studies, it is difficult to identify factors unique to groups of South Asian women based on nationality, geographical region or religion.
- High quality research on screening attendance is required using prospective designs.

For peer review only

For peer review only - http://bmjopen.bmj.com/site/about/guidelines.xhtml

Introduction

Since 1945, many countries have benefited economically and socially from large-scale migration from the South Asian nations of India, Pakistan, Bangladesh, Sri Lanka, Nepal, Maldives and Bhutan [1]. Migration has largely favoured English-speaking countries, although large South Asian populations also exist in European, African and neighbouring Asian countries [2]. In the United Kingdom (UK), the South Asian population constitutes the largest ethnic minority category [3]. In all host countries, historic migration patterns have led to the establishment of South Asian communities in cities and large towns where cultural norms and practices of the countries of origin are practised alongside those of the host country [4]. In the UK, with the exception of some Indian groups, South Asian women have worse cancer-related health outcomes compared with the host population [3].

South Asian women in the UK have higher breast and cervical cancer mortality and are more likely to present with advanced disease [5]. This is partly attributable to their lower likelihood of attending routine mammographic and Papanicolaou (Pap) screening. Screening is widely available in most high income countries [6-8] and probably reduces mortality and morbidity through early detection and treatment [9]. South Asian women in England show lower uptake of breast screening services than the host population [10-13], particularly those from lower socioeconomic groups [10 14 15] and a higher proportion than the host population have never received cervical screening [16].

It is unclear why screening rates in South Asian women are lower than host population rates. Possible explanations have included poorer individual knowledge and awareness of breast and cervical cancer [17-19], lower community awareness, poor communication between health professionals and patients and less access to appropriate cancer health services [20 21]. Some South Asian women cannot speak or read in the host language [22 23]. Another body of research focuses on South Asian women's attitudes, beliefs

For peer review only - http://bmjopen.bmj.com/site/about/guidelines.xhtml

and behaviours relating to cancer screening [24 25]. Crawford et al. [25] and Sokal [22] recently compiled scoping and critical reviews of breast, cervical and colorectal screening in South Asian populations in Canada, the UK and the USA. The reviews demonstrate how individuals' beliefs, knowledge and perceptions of access barriers are shaped by the host environment, migration experience, cultural references and practices of the country of origin, and the cultural processes of adaptation to the host country.

Both Crawford et al. and Sokal's reviews have limitations. Both combined studies with differing methodological approaches to achieve integrated descriptions of findings. This approach may lead to bias because it does not separate content from method [26], leading to two limitations. First, critical examination of study quality is more difficult when varying methodologies are used. Thus, the value of findings cannot be moderated or weighted by quality appraisal of the reviewed studies and it is not possible to critique current methods to recommend better approaches. Second, the reader cannot determine if insights are confined to a single method or are replicated across different approaches.

Whittemore and Knafl [27] describe a method of integrative review that resolves these problems by separately integrating findings within different methodologies, then comparing the integrative findings across analyses to identify consistencies and limitations of findings within and between methods. Researchers into migrant health use four basic types of investigation. *Inductive* studies use qualitative analyses that allow participants to present their own experiences, thus providing novel insights that drive theory development. *Predictive, comparative* and *intervention* studies are deductive, using quantitative methods to test hypotheses. *Predictive* studies predict health behaviour from measures of individual and contextual attributes, allowing theory testing by quantifying associations between predictors and outcomes within migrant populations. *Comparative* studies compare target populations with host or other immigrant populations to identify whether the determinants of health

For peer review only - http://bmjopen.bmj.com/site/about/guidelines.xhtml

behaviour in immigrant groups are unique to them or are shared with host or other immigrant groups. Shared factors include relative economic deprivation [22 28] or social and cultural adjustment challenges [29]. It is also important to review reports of *intervention* studies to examine how successful previous interventions (or their individual components) have been in improving screening rates in South Asian populations.

Aims of the Review

We examined cultural, social, structural and behavioural factors that influence asymptomatic breast and cervical cancer screening in South Asian populations, to explain why attendance rates are lower than host country women. We performed separate integrative reviews of inductive, predictive, comparative and intervention studies, and compared outcomes of these reviews to assess consistencies between methods. Our aim was to identify the cultural, social, structural and behavioural factors that influence asymptomatic breast and cervical cancer screening rates in South Asian populations to improve screening rates and to propose priorities for further research. Our objectives were to:

- critically review and integrate findings of inductive, predictive, comparative and intervention studies on asymptomatic screening;
- document consistent and inconsistent findings across methods;
- make theoretical and methodological recommendations for the conduct of future research.

For peer review only

For peer review only - http://bmjopen.bmj.com/site/about/guidelines.xhtml

Methodology

Search strategy

We conducted literature searches using multiple databases to overcome problems associated with inadequate indexing [27 30] and to ensure a more exhaustive scope [27 31 32]. We searched the following databases: MEDLINE/In-Process; Web of Science; EMBASE; SCOPUS; CENTRAL; CDSR; CINAHL; PsycINFO and PsycARTICLES for 4 key concepts: 1) South Asian population 2) cancer 3) asymptomatic breast or cervical screening and 4) knowledge, attitude, practice, behaviour or compliance. PubMed was searched for publications ahead of print and conference proceedings. Search terms were revised after initial searches revealed new terms. MeSH terms were run in combination with free-text searches of titles and abstracts. These are available as an online data supplement at https://www.crd.york.ac.uk/PROSPEROFILES/25284_STRATEGY_20170702.pdf. . Searches were conducted from database inception to 12th July 2016. The search was restricted to original research in English for all publication dates. Citations of selected studies were reviewed to identify any additional studies. We checked for grey literature via databases and repositories such as Open SIGL, Open Grey, PsycEXTRA, HMIC UK, The Grey Literature Report, ClinicalTrials.gov, NTIS, NCIN and the WHO ICTRP, and cancer and clinical networks including American Cancer Society, South Asian Health Foundation and MacMillan Cancer Support.

Selection criteria

The review included studies of attendance at asymptomatic screening of female breast and/or cervical cancers by routine mammography and cervical smear testing (Papanicolaou test). It was confined to host countries where mass screening programmes are available to the general public. The populations of interest were Bangladeshi, Indian, Pakistani, Sri Lankan,

For peer review only - http://bmjopen.bmj.com/site/about/guidelines.xhtml

Bhutanese, Maldivian and Nepali populations (or ethnic subgroups thereof). Studies needed to report on samples or subsamples identifiable as wholly South Asian.

The review did not include breast self-examination, diagnostic screening or visual or tactile examinations by healthcare professionals. We excluded studies of women in known high-risk groups who were engaged in monitoring programmes for genetic risk factors, hereditary breast and ovarian cancer syndrome, premenopausal or familial breast cancer. We excluded homogenous samples restricted to particular demographic groups (e.g. a study of dental students).

Screening

Team members screened titles and abstracts to identify potentially eligible studies and two reviewers independently considered the eligibility of each of the titles and abstracts. Outputs were compared to detect discrepancies and the agreement rate was 90%. Disagreements over selection of abstracts were resolved by consensus between the team. Calibration of the selection criteria was performed after the first 50 and 100 papers and taking a small sample (15%) of reports from grey and unpublished literature. Two reviewers independently assessed the full text of relevant studies using a standardized, pilot-tested screening form agreed with the steering group. Disagreements were resolved by consensus or by referral to a third-party arbiter. EndNote (X5) reference manager was used to manage citations and view abstracts and full-text articles.

Quality evaluation

Each study was evaluated for quality specific to the method used, with validated checklists developed from the Critical Appraisal Skills Programme [33]. Inductive studies were generally found to be good. Predictive, descriptive and intervention studies had theoretical, sampling,

For peer review only - http://bmjopen.bmj.com/site/about/guidelines.xhtml

design and measurement limitations. We did not exclude studies that used poor methodologies, but extensively describe these problems and consequent interpretive limitations in the results.

Data extraction, synthesis and analysis

All studies included in the review are included in summary tables (Tables 1 to 4). Four reviewers completed data extraction for each study type and reviewed the variable headings upon completion [34]. Subsequently, tables were adapted and the following variables were recorded for all studies: region, study design (sample size and sampling); demographic and clinical characteristics of women selected; setting; data collection instruments; analytic method; nature of asymptomatic screening (mammogram or Pap smear test); definition of timely screening attendance; theoretical focus; key findings; study limitations and quality rating. For predictive studies we recorded outcome variables, rate of screening attendance and all predictors for and against screening. Intervention studies included a description of the intervention concerned.

Syntheses were made using thematic analysis within each methodology type [27 35]. Syntheses were initially structured from the summary tables, beginning with a period of data familiarisation, during which researchers listed ideas about emerging themes which formed the basis of a thematic framework. At this point, the analysis returned to the full papers, where the developing thematic framework was tested and refined against the initial data. Themes were developed, reviewed and refined by analysing the data synthesised within each code and testing for 'internal homogeneity' and 'external heterogeneity' [36]. The research group met continuously to check and discuss the meaning and interpretation of the data.

For peer review only - http://bmjopen.bmj.com/site/about/guidelines.xhtml

Results

The combined search of electronic bibliographic databases yielded 8737 citations (Figure 1). After removing duplicates (n = 3064), the remaining 5673 were screened on title and 1040 on abstract. 103 records were selected for full-text review. Subsequently, 60 were eliminated and 43 included in the review syntheses. The 43 studies were published between 1991 and 2016 and were conducted in the USA (n=19), Canada (n=12), UK (n=5), Spain (n=2), Singapore (n=2), Malaysia (n=2) and Australia (n=1). Sample sizes ranged from 25 to 38,733. Participants were recruited from community and healthcare settings and had a mean age of 18 to 83 years. Eight were inductive, 21 predictive (containing analysis of predictors of and risk factors for attendance), 9 comparative, and 6 intervention studies. No further studies were found from the grey literature search.

Overview

Inductive studies provided rich insights into cultural practices and assumptions, and the problems of adjusting to a new social and healthcare system that might inhibit screening in South Asian women. Largely, though, deductive studies failed to exploit these insights in hypothesis testing. Deductive studies were either atheoretical or used generic health psychology theories, such as the health belief model, that were validated on western samples and not adapted for South Asian populations.

Nonetheless, common findings emerged across methodologies. The extent to which women understood the causes of cancer and the benefits of screening were important. Inductive studies revealed cultural constraints on understanding, whilst comparative studies showed South Asian women faring worse on measures of knowledge than host country women. Predictive studies showed that those with more complete understandings of cancer and screening were more likely to attend screening. Similarly, both inductive and deductive

For peer review only - http://bmjopen.bmj.com/site/about/guidelines.xhtml

studies showed that perceived barriers inhibited screening, and that South Asian women typically perceived more and different barriers to host country women. Inductive studies showed the cultural origins of barriers, describing how traditional beliefs about risk, illness, female roles and family structures mitigated screening interest and attendance. Predictive studies showed that the number of perceived barriers inhibited screening, and that South Asian women who were more acculturated to western host countries, operationalized as time spent in those countries, were more likely to attend screening.

Inductive studies

The seven inductive studies (Table 1) were conducted in Canada and the USA, amongst Pakistani, Indian, Sri-Lankan Tamil and Bhutanese populations. Sample sizes ranged from 27-68, with a median of 43. Women had varying lengths of residency and were mostly born outside the host country. Six studies [37-42] used in-depth interviews and/or focus group discussions. One study [43] employed concept mapping using participatory research methods. Pons-Vigues et al. acknowledged difficulties in interviewing women whose cultural backgrounds differed most from their own, and championed the need for cultural intermediaries. However, intermediaries were not used in the other studies. Studies focussed on the experiences of women themselves, and did not interview family members or health care providers.

Data synthesis generated three overarching themes: '*Knowledge, attitudes, understanding of cancer and cancer prevention*; '*Culture*'; and *'The process of Cultural adaptation'* to the host country.

For peer review only - http://bmjopen.bmj.com/site/about/guidelines.xhtml

Knowledge, attitudes, understanding of cancer and cancer prevention: Neither cancer nor intimate body parts are commonly discussed in some South Asian cultures [38 42]. All studies showed that women lacked basic understandings of cancer, cancer prevention or early detection. Breast cancer was viewed by some women as a '*white woman's disease'* [37], that did not occur in their community [41]. Others considered cancer to be incurable and early detection and intervention futile [41]. Cervical cancer was often not known or understood. For example, some Bhutanese refugees in the USA had not heard of cervical cancer [41]. Indian Sikhs in Canada, living in a culture where sexual and reproductive health is rarely discussed, referred to the cervix as an 'unknown' and unspoken part of their body [42]. Those aware of cervical cancer perceived the principal risk factors to be inseparable from those for general health, rarely mentioning the discrete risk factors of having multiple sexual partners, not using barrier contraception or screening [41].

Studies attributed a lack of understanding of cancer to two main factors. First, religious fatalism meant that cancer was seen as predestined, as divine retribution for sins, or as a dearth of moral character [38]. Second, all studies pointed to the curative focus of healthcare in countries of origin as a reason for some women's failure to understand the concept of prevention [38 44] and consequent belief that healthcare seeking is unnecessary in the absence of symptoms [37 40 42].

Culture: Family responsibilities were salient to women. This had three implications, one positive and two negative. First, women felt strong responsibilities to remain in good health and to protect family members from cancer [40]. This facilitated screening attendance. Second, notions of stigma precluded screening. Themes of 'shyness', 'modesty' and 'embarrassment' about revealing intimate body parts were important [37]. For example, Bhutanese refugees worried that attending a Pap test would damage reputations for chastity [41]. Both breast and

For peer review only

For peer review only - http://bmjopen.bmj.com/site/about/guidelines.xhtml

cervical cancer were seen as stigmatising [42] and to some women this extended to screening [38 41]. Indeed, some Sri Lankan Tamils worried that attending a mammogram would lead people to think they already had breast cancer [38]. Third, women's behaviour was often subject to influence from male members of the family. Women frequently followed family advice for healthcare provided by males and elders, generally against screening, and felt the need to avoid conflict within the family associated with assertions of independence [37 42].

The process of Cultural Adaptation: South Asian nations have largely curative health systems, in which health costs are required to be paid by patients and there is no free access to healthcare. This contrasts with preventive healthcare models in host countries, and as with other health issues South Asian women showed little understanding or orientation toward cancer prevention [42], although this evolved with time as awareness of the culture of the host country increased [39 43]. Women appreciated healthcare professionals who understood and respected values of personal modesty/shyness [41]. South Asian women in Canada emphasised the value of being chaperoned to screening appointments that may have been located away from their local community, for assistance with language barriers, to alleviate feelings of personal vulnerability and to avoid being alone with doctors [37].

Deductive Studies

Study Quality: Predictive and comparative studies contained similar limitations to quality. The first limitation was the poverty of theory. With the exception of Pons-Vigues and colleagues, whose deductive study [45] was informed by their earlier inductive work [39], we noted little correspondence between inductive themes and hypotheses tested in the deductive research. Studies focussed on knowledge of cancer and screening, but were not informed by themes of fatalism, non-understanding of preventive healthcare, or cultural and family

For peer review only - http://bmjopen.bmj.com/site/about/guidelines.xhtml

systems found in the qualitative research. Instead, studies were theoretically based upon western health behaviour theories, such as the health belief model (HBM) [46], with limited applicability to South Asian populations. Similarly, the concept of acculturation was invoked in predictive studies, but was operationalised in a limited way, focussing on time spent in the emigrant country and language preferences. Other deductive work was not theoretically-based.

Studies were also affected by methodological limitations. A database linkage study [47] and two cluster sampling studies [48 49] provided samples with a potentially high degree of population representativeness, with random digit dialling techniques providing some confidence that samples may be representative [50 51]. Other studies used poor sampling techniques, including selection of South Asian names from phone directories or sampling at cultural events or other locations with high proportions of South Asian women, providing less confidence. Definition of a South Asian population differed between studies, some examined women born in South Asia, others second generation immigrants, and some examined self-identified ethnicity.

It is important that attendance is recorded objectively [52]. All studies but one [47] used non-verified self-reported attendance and one used a hypothetical scenario of an offer to attend screening [50]. These outcomes included timely screening attendance (e.g., previous screening was within a specified time period or reported regular timely testing) or whether women had ever been screened in the past.

<u>Predictive Studies:</u> It is strongly recommended that predictive studies be conducted prospectively to eliminate the problem of reverse causality [52]. All of the 21 predictive studies were cross-sectional and causal interpretation is difficult.

For peer review only - http://bmjopen.bmj.com/site/about/guidelines.xhtml

Lower screening rates were noted among women with no health insurance, younger women and women with lower levels of education. Studies did not provide consistent evidence that low knowledge predicted reduced likelihood of attendance. Lower knowledge was associated with a reduced likelihood of mammography screening in two studies [53 54], but did not predict the likelihood of hypothetical acceptance of a cervical screen [50]. Lower attendance was associated with a greater number of self-reported barriers to screening [53-57]. However, the instruments used to assess barriers were largely based upon existing instruments developed among western samples that do not reflect South Asian concerns such as adapting to a new culture, language or health system.

Where acculturation was examined, less time spent in the host country was the strongest predictor of non-attendance, although one study cited lower preference for the host language (usually English) compared to women's native language [57]. Vahabi, et al. [47] found that South Asian women were less likely to attend mammography screening if their GP had qualified outside the host country.

Comparative Studies: Eight of the nine comparative studies compared South Asian women with host populations, eight compared South Asian women to other minority groups. South Asian samples often differed from comparison samples on demographic variables such as socioeconomic status (from lower socioeconomic backgrounds) and relationship status (mostly married), which limits trust that can be placed on comparisons if these factors are not statistically adjusted for.

Three comparisons with host populations showed South Asian women to have lower screening rates [48 49 58], but two did not [59 60]. Of these studies, Dunn and Tan and Marlow et al. used sampling techniques more likely to derive representative samples. Lower screening rates

For peer review only - http://bmjopen.bmj.com/site/about/guidelines.xhtml

may be attributable to the knowledge deficits and greater perceived barriers observed in some studies [45 49 61].

Two methodologically rigorous comparisons between South Asian and other minority groups [47 48] used population sampling and statistically adjusted for demographic differences between samples. Vahabi, et al [47] also used an objectively verified indicator of mammography attendance. Both showed South Asian women to have lower attendance rates than other immigrant women. In two studies, Indian women had lower knowledge of cancer and screening than Chinese or Malays [48 61]. Pons-Vigues et al. [45] and Teo et al [58] showed Indian and Pakistani women perceived fewer barriers arising from lack of knowledge about preventative screening than other immigrant groups, and highlighted that many of the women thought that routine blood tests and urine tests would detect broader health issues such as cancer [45]. In another study [59], Indian women perceived themselves to be less vulnerable to getting breast cancer, did not view breast cancer as a serious illness and were more likely to claim that they did not know 'where to find a mammogram'.

<u>Intervention Studies</u>: Community educational programmes promoted breast and cervical cancer screening across the six intervention studies. Three of the studies were pre-and post-community-based interventions [62-64], two were randomised control trials [44 65]; and one a time series study [66]. Sampling was predominantly among South Asian women as a group, which eliminates comparisons between the different South Asian populations. Studies employed various methods of socioculturally-tailored, language-specific health education materials and participants were recruited from primary care or South Asian community venues and residences. Recruitment was opportunistic via local newspapers, surveys conducted in community settings, South Asian nurses and link health workers. No study examined age trends [64]; and participants had met the researchers before which may constitute a bias [44].

For peer review only - http://bmjopen.bmj.com/site/about/guidelines.xhtml

Controlled studies were conducted in close-knit communities which may have led to intervention contamination into the control groups. Increased screening rates were reported for three studies but many were self-reported [62 63]; rather than from objective indicators [44]. No long-term change in screening uptake was reported for three studies [64-66], but they showed an increase in knowledge of breast cancer among South Asian immigrant women and reduced the misperception of short survival after diagnosis.

For peer review only

For peer review only - http://bmjopen.bmj.com/site/about/guidelines.xhtml

Discussion

Prominent across study types were the findings that South Asian women had poorer understandings of cancer and cancer prevention and that they perceived greater cultural and structural barriers to screening than host country women.

Lack of understanding by South Asian women about the need for asymptomatic screening has important ramifications. Predictive studies showed greater knowledge to be associated with screening attendance. The inductive research yielded some plausible reasons for this. Many women held fatalistic views or beliefs that cancer is incurable, whilst others believed that cancers could be identified in routine health testing. Others were unaware of the existence of cervical cancer in particular, and did not perceive threat to themselves or their communities. Whilst there is a clear need to change such beliefs, the inductive studies showed this to be a challenging task for two reasons. First, understandings were embedded within religious and cultural traditions, and cannot be addressed in isolation to those traditions. Thus, a simple educational intervention is likely to have limited effect. Accommodations will need to be reached with communities that allow a creative integration of cancer awareness within existing belief structures. Second, some women were largely unaware of the concept of disease prevention. Thus, the promotion of specific cancer awareness and understandings are unlikely to be helpful until a wider understanding of prevention is reached.

Predictive studies showed the importance of perceived barriers, but these barriers pertained only to generic barriers faced by all women, irrespective of culture. Acculturation, in terms of time spent in the host country and mastery of the language was associated with increased screening likelihood, but these issues are likely to exist for all immigrant women and fail to reveal specifically South Asian issues. Inductive studies provided more subtle and culture-specific indications of the barriers perceived by women. Many were cultural. In

For peer review only - http://bmjopen.bmj.com/site/about/guidelines.xhtml

particular, women spoke of the importance of female modesty and stigma associated with cancer that also affected willingness to be screened. Whilst the importance of female testing staff from South Asian backgrounds and use of South Asian chaperones is emphasised, this cannot address the wider cultural issues of modesty and stigma. One finding that offers encouragement is that personal health is important to South Asian women because it helps them to care for their families.

Interventions will need to be conducted more widely than merely targeting women and their beliefs. Males occupy decision-making roles in some South Asian families and women may not wish to challenge this (see also [67 68]. Thus, addressing the views of male family members and other community opinion leaders is also important.

Limitations

The following limitations were identified within the review. First, studies were primarily conducted in Canada and the USA. North American conditions, such as payment-oriented healthcare and whether screening is promoted by a central agency or not, are not necessarily replicable in other health systems. Second, due to the small number of published studies, it is difficult to identify factors unique to groups of South Asian women based on nationality, geographical region or religion. By necessity, we discuss findings in terms of a generic 'South Asian' population, but are aware of variance between South Asian populations according to nationality, region, culture and religion.

Future Research

Stratifying the analysis by study methodology brings two benefits; greater confidence can be placed on findings that transcend methodologies than those that are contained within one method, and studies with similar methodologies can be critiqued in ways appropriate to those

For peer review only - http://bmjopen.bmj.com/site/about/guidelines.xhtml

methodologies. This review emphasises the generally poor quality of the deductive literature. Failure to incorporate inductive findings into the design of deductive studies means that many inductive findings are untested in a population context. Further, deductive studies themselves used flawed designs as they were generally atheoretical or based upon health behaviour models developed in western populations and thus potentially lacking insight into South Asian issues. Translation of inductive findings to a deductive context will require the development of valid and reliable instruments to assess cultural understandings, beliefs, norms and practices.

These studies will also need to use better empirical methods. Few studies used sampling techniques that can be confidently claimed to be population-representative. It is important to employ best practice in study design for screening attendance research; the use of prospective predictive studies and objectively verified reporting of attendance from clinical records [52]. Adequate sampling frames need to be established. Firstly, this involves a distinction between South Asian women as a minority group or as an immigrant group. The former can comprise women with high degrees of familiarity with the host country, but who nonetheless may be faced with cultural barriers deriving from their countries of origin. The latter group will reflect the problems of adjustment faced by recent immigrants. Studies will also need to use population-representative sampling techniques.

Recommendations for Practice

Findings from all study types demonstrate that interventions should be sensitive to cultural norms. In particular, studies emphasised the importance of language, female practitioners and the importance of community approval and involvement. Interventions at the community level will be necessary to surmount the cultural barriers identified in the inductive studies.

For peer review only - http://bmjopen.bmj.com/site/about/guidelines.xhtml

It is worrying that the findings indicated that younger women and women with lower levels of education were less likely to attend for screening. Interventions need to be targeted at educating South Asian women who are younger, not married and less educated. Encouraging female family members to become more involved as chaperones and translators could also be helpful, and may form a mechanism for educating young women simultaneously.

Information aimed at South Asian women who are invited for breast and cervical screening should highlight the presence of female practitioners and exclusively female environments at breast and cervical screening sites in the UK [69]. There is limited use of written communication in South Asian languages, although 70% of screening units across the UK want to provide information in patient's language [70]. This may help improve South Asian women's knowledge, make informed choice/ consent, have better patient experience and eventually help in improving their screening uptake rates.

Interventions to increase uptake rates need to be long-term, multifaceted and tailored to the specific needs of the local community by, for example, developing close links with the community through Health Education workers. South Asian community members, including males and opinion leaders, should be encouraged to be involved and co-produce engagement strategies within community settings. Reducing ethnic inequalities in uptake rates of breast cancer screening needs to remain a policy priority of the Government and the Breast Screening Programme.

Funding

PS and SB are funded by the National Institute for Health Research Collaboration for Leadership in Applied Health Research and Care North West Coast (NIHR CLAHRC NWC) (Grant/Award Number: CLAHRC-NWC-015).

For peer review only - http://bmjopen.bmj.com/site/about/guidelines.xhtml

Acknowledgements

The authors thank Professor Andy Clegg, the Royal Liverpool and Broadgreen Hospital Trust, the PREVENT Breast Cancer Charity in Manchester and the South Asian Women who contributed to the study at public engagement events.

Contributions

P.S. and S.B. designed this study; P.S., S.B., R.AdC., D.R., N.T., S.A., K.B., A.J., M.C. and E.K. contributed in finalising search terms; R.AdC., E.K. and D.R. searched databases and R.AdC. and D.R. collected full-text papers; P.S., S.B., R.AdC. and D.R. extracted and analyzed data; N.T. and S.A. contributed with reviewing abstracts, reading full text papers and extracting data. P.S., R.AdC. and S.B. wrote the manuscript, K.B. reviewed the manuscript.

Competing Interests

The views expressed are those of the author(s) and not necessarily those of the NHS, the NIHR or the Department of Health.

Conflicts of Interest

The Authors declare no conflict of interest.

Data sharing

No additional data available.

For peer review only - http://bmjopen.bmj.com/site/about/guidelines.xhtml

References

1. ONS. Cancer Registration Statistics , England: 2013: Office for National Statistics, 2013
2. UN. Human Development Reports. United Nations Development Programme, 2008
3. ONS. Cancer Registration Statistics, England: 2011: Office for National Statistics, 2011.
4. Ballard R. The South Asian presence in Britain and its transnational connections. In: Singh H, Vertovec S, eds. Culture and economy in the Indian diaspora. London: Routledge 2002:197-222.
5. Forbes LJ, Atkins L, Thurnham A, et al. Breast cancer awareness and barriers to symptomatic presentation among women from different ethnic groups in East London. *Br J Cancer* 2011;105(10):1474-79.
6. WHO. WHO Guidance Note. Comprehensive cervical cancer prevention and control: A healthier future for girls and women 2013 [Available from: http://apps.who.int/iris/bitstream/10665/78128/3/9789241505147_eng.pdf.
7. WHO. Position Paper on Mammography Screening 2014 [Available from: http://www.who.int/cancer/publications/mammography_screening/en/ accessed 19th August 2015 2015.
8. WHO. Breast Cancer: prevention and control 2015 [Available from: http://www.who.int/cancer/detection/breastcancer/en/ accessed 25 August 2015 2015.
9. Yip C-H, Smith RA, Anderson BO, et al. Guideline Implementation for Breast Healthcare in Low- and Middle-Income Countries: Early Detection Resource Allocation. *Supplement to Cancer* 2008
10. Hoare T. Breast screening and ethnic minorities. *British Journal of Cancer* 1996;74(Supplement XXIX):S38 - S41.
11. Waller M, Moss S, Watson J, et al. The effect of mammographic screening and hormone replacement therapy use on breast cancer incidence in England and Wales. *Cancer epidemiology, biomarkers & prevention : a publication of the American Association for Cancer Research, cosponsored by the American Society of Preventive Oncology* 2007;16(11):2257-61. doi: 10.1158/1055-9965.EPI-07-0262 [published Online First: 2007/11/17]
12. Moser K, Patnick J, Beral V. Inequalities in reported use of breast and cervical screening in Great Britain: analysis of cross sectional survey data. *Bmj* 2009;338:b2025. doi: 10.1136/bmj.b2025 [published Online First: 2009/06/18]
13. Robb K, Power E, Atkin W, et al. Ethnic differences in participation in flexible sigmoidoscopy screening in the UK. *Journal of Medical Screening* 2008;15:130-36. doi: 10.1258/jms.2008.007112
14. Edmundson C, Jain A, Astley S. The impact of socioeconomic factors and ethnicity on breast screening uptake in the North West of England. Poster. *British Journal of Radiology* 2009;Supplements
15. Tollitt J, Jain A, Astley S. Health inequalities in breast cancer screening. *Breast Cancer Research* 2008;Suppl 2 (10):1. doi: doi:10.1186/bcr1941
16. Webb R, Richardson J, Esmail A, et al. Uptake for cervical screening by ethnicity and place-of-birth: a population-based cross-sectional study. *Journal of public health* 2004;26(3):293-6. doi: 10.1093/pubmed/fdh128 [published Online First: 2004/09/30]
17. Scanlon K, Wood A. Breast cancer awareness in Britain: are there differences based on ethnicity? *Diversity in Health & Social Care* 2005;2(3)
18. DH. Cancer Reform Strategy: Achieving local implementation - second annual report. NHS. In: DoH, ed.: www.dh.gov.uk/publications, 2009.
19. Johnson C, Mues K, Mayne S, et al. Cervical cancer screening among immigrants and ethnic minorities: a systematic review using the Health Belief Model. *J Low Genit Tract Dis* 2008;12:232-41.

For peer review only - http://bmjopen.bmj.com/site/about/guidelines.xhtml

20. Zaman MJS, Mangtani P. Changing disease patterns in South Asians in the UK. *Journal of the Royal Society of Medicine* 2007;100(June):254-55.
21. Elkan R, Avis M, Cox K, et al. The reported views and experiences of cancer service users from minority ethnic groups: a critical review of the literature. *European Journal of Cancer Care* 2007;16:109-21.
22. Sokal R. A critical review of the literature on the uptake of cervical and breast screening in British South Asian women. *Qual Prim Care* 2010;18(4):251-61.
23. Jain A. UK: South Asian Women at the Crossroads NHS Evidence Ethnicity and Health ed, 2010.
24. Wu TY, Guthrie BJ, Bancroft JM. An integrative review on breast cancer screening practice and correlates among Chinese, Korean, Filipino, and Asian Indian American women. *Health Care Women Int* 2005;26(3):225-46.
25. Crawford J, Ahmad F, Beaton D, et al. Cancer screening behaviours among South Asian immigrants in the UK, US and Canada: a scoping study. *Health & social care in the community* 2016;24(2):123-53. doi: 10.1111/hsc.12208 [published Online First: 2015/02/28]
26. Beck CT. Facilitating the work of a meta-analyst. *Research in nursing & health* 1999;22(6):523-30. [published Online First: 2000/01/08]
27. Whittemore R, Knafl K. The integrative review: updated methodology. *Journal of Advanced Nursing* 2005;52(5):546-53.
28. Szczepura A, Price C, Gumber A. Breast and bowel cancer screening uptake patterns over 15 years for UK south Asian ethnic minority populations, corrected for differences in socio-demographic characteristics. *BMC Public Health* 2008;8:346. doi: http://dx.doi.org/10.1186/1471-2458-8-346
29. Marlow L, Wardle J, Waller J. Understanding cervical screening non-attendance among ethnic minority women in England. *British Journal of Cancer* 2015;113:833-39. doi: 10.1038/bjc.2015.248
30. Cooper H. Synthesizing Research: A Guide for Literature Reviews. Second ed. California: Sage Publications 1998.
31. Counsell C. Formulating questions and locating primary studies for inclusion in systematic reviews. *Annals of Internal Medicine* 1997;127:380-87.
32. Jackson N, Waters E. Criteria for the Systematic Review of Health Promotion and Public Health Interventions. *Health Promotion International* 2005;20(4):367-74.
33. CASP. Critical Appraisal Skills Programme (CASP) Checklist Oxford: CASP; 2006 [Available from: www.casp-uk.net/ accessed 1 October 2015.
34. Moher D, Liberati A, Tetzlaff J, et al. Preferred Reporting Items for Systematic Reviews and Meta-Analyses: The PRISMA Statement. *PLoS Med* 2009;6(7) doi: 10.1371/journal.pmed.1000097
35. Braun V, Clarke V. Using thematic analysis in psychology. *Qualitative research in psychology* 2006;3(2):77-101.
36. Patton MQ. Qualitative evaluation and research methods, 2nd ed. Thousand Oaks, CA, US: Sage Publications, Inc 1990.
37. Bottorff JL, Johnson JL, Bhagat R, et al. Beliefs related to breast health practices: the perceptions of South Asian women living in Canada. *Soc Sci Med* 1998;47(12):2075-85.
38. Meana M, Bunston T, George U, et al. Older immigrant Tamil women and their doctors: attitudes toward breast cancer screening. *J immigr health* 2001;3(1):5-13.
39. Pons-Vigues M, Puigpinos-Riera R, Rodriguez D, et al. Country of origin and prevention of breast cancer: beliefs, knowledge and barriers. *Health Place* 2012;18(6):1270-81. doi: http://dx.doi.org/10.1016/j.healthplace.2012.09.008
40. Bottorff JL, Balneaves LG, Sent L, et al. Cervical cancer screening in ethnocultural groups: case studies in women-centered care. *Women Health* 2001;33(3-4):29-46.
41. Haworth R, Margalit R, Ross C, et al. Knowledge, Attitudes, and Practices for Cervical Cancer Screening Among the Bhutanese Refugee Community in Omaha, Nebraska. *J Community Health* 2014;39(5):872-78 7p. doi: 10.1007/s10900-014-9906-y

For peer review only - http://bmjopen.bmj.com/site/about/guidelines.xhtml

42. Oelke ND, Vollman AR. "Inside and outside": Sikh women's perspectives on cervical cancer screening. *Can J Nurs Res* 2007;39(1):174-89.
43. Ahmad F, Mahmood S, Pietkiewicz I, et al. Concept mapping with South Asian immigrant women: barriers to mammography and solutions. *J Immigr Minor Health* 2012;14(2):242-50. doi: http://dx.doi.org/10.1007/s10903-011-9472-7
44. McAvoy BR, Raza R. Can health education increase uptake of cervical smear testing among Asian women? *BMJ (Clinical research ed)* 1991; 302(6780). http://onlinelibrary.wiley.com/o/cochrane/clcentral/articles/153/CN-00075153/frame.html.
45. Pons-Vigues M, Puigpinos-Riera R, Serral G, et al. Knowledge, attitude and perceptions of breast cancer screening among native and immigrant women in Barcelona, Spain. *Psychooncology* 2012;21(6):618-29. doi: http://dx.doi.org/10.1002/pon.1940
46. Becker M. The Health Belief Model and personal health behavior. *Health Education Monographs* 1974;2:324-508.
47. Vahabi M, Lofters A, Kumar M, et al. Breast cancer screening disparities among urban immigrants: a population-based study in Ontario, Canada. *BMC Public Health* 2015;15:679. doi: http://dx.doi.org/10.1186/s12889-015-2050-5
48. Dunn RA, Tan AK. Cervical cancer screening in Malaysia: Are targeted interventions necessary? *Soc Sci Med* 2010;71(6):1089-93. doi: http://dx.doi.org/10.1016/j.socscimed.2010.06.016
49. Marlow LA, Wardle J, Waller J. Understanding cervical screening non-attendance among ethnic minority women in England. *Br J Cancer* 2015;113(5):833-9. doi: http://dx.doi.org/10.1038/bjc.2015.248
50. Chawla N, Breen N, Liu B, et al. Asian American women in California: a pooled analysis of predictors for breast and cervical cancer screening. *Am J Public Health* 2015;105(2):e98-e109. doi: http://dx.doi.org/10.2105/AJPH.2014.302250
51. Lin MK, Moskowitz JM, Kazinets G, et al. Adherence to pap test guidelines: Variation among Asians in California. *Ethnicity and Disease* 2009;19(4):425-32.
52. Sabatino SA, Lawrence B, Elder R, et al. Effectiveness of interventions to increase screening for breast, cervical, and colorectal cancers: nine updated systematic reviews for the guide to community preventive services. *American journal of preventive medicine* 2012;43(1):97-118. doi: 10.1016/j.amepre.2012.04.009 [published Online First: 2012/06/19]
53. Hasnain M, Menon U, Ferrans CE, et al. Breast cancer screening practices among first-generation immigrant muslim women. *J Womens Health (Larchmt)* 2014;23(7):602-12. doi: http://dx.doi.org/10.1089/jwh.2013.4569
54. Marfani F, Rimal RN, Juon H-S. Understanding immigrant women's information needs: Role of acculturation in breast cancer prevention among immigrant Asian Indian women. *J Appl Commun Res* 2013;41(2):126-40. doi: 10.1080/00909882.2012.754485
55. Ahmed NU, Fort JG, Elzey JD, et al. Empowering factors in repeat mammography: Insights from the stories of underserved women. *J Ambulatory Care Manage* 2004;27(4):348-55.
56. Meana M, Bunston T, George U, et al. Influences on breast cancer screening behaviors in Tamil immigrant women 50 years old and over. *Ethn Health* 2001;6(3-4):179-88.
57. Menon U, Szalacha LA, Prabhughate A. Breast and cervical cancer screening among South Asian immigrants in the United States. *Cancer Nurs* 2012;35(4):278-87. doi: http://dx.doi.org/10.1097/NCC.0b013e31822fcab4
58. Teo CT, Yeo YWS, Lee SC. Screening mammography behavior and barriers in Singaporean Asian women. *Am J Health Behav* 2013;37(5):667-82. doi: 10.5993/AJHB.37.5.11
59. Wu TY, West B, Chen YW, et al. Health beliefs and practices related to breast cancer screening in Filipino, Chinese and Asian-Indian women. *Cancer Detect Prev* 2006;30(1):58-66.
60. Wu TY, Hsieh HF, West BT. Demographics and perceptions of barriers toward breast cancer screening among Asian-American women. *Women Health* 2008;48(3):261-81. doi: http://dx.doi.org/10.1080/03630240802463384

For peer review only - http://bmjopen.bmj.com/site/about/guidelines.xhtml

61. Abdul Hadi M, Hassali MA, Shafie AA, et al. Knowledge and perception of breast cancer among women of various ethnic groups in the state of Penang: a cross-sectional survey. *Med Princ Pract* 2010;19(1):61-7. doi: http://dx.doi.org/10.1159/000252837
62. Ahmad F, Cameron JI, Stewart DE. A tailored intervention to promote breast cancer screening among South Asian immigrant women. *Soc Sci Med* 2005;60(3):575-86.
63. Kernohan EE. Evaluation of a pilot study for breast and cervical cancer screening with Bradford's minority ethnic women; a community development approach, 1991-93. *Br J Cancer Suppl* 1996;29:S42-6.
64. Sadler GR, Ryujin L, Nguyen T, et al. Heterogeneity within the Asian American community. *Intern* 2003;2:1-9. doi: http://dx.doi.org/10.1186/1475-9276-2-1
65. Hoare T, Thomas C, Biggs A, et al. Can the uptake of breast screening by Asian women be increased? A randomized controlled trial of a linkworker intervention. *J Public Health Med* 1994;16(2):179-85.
66. Grewal S, Bottorff JL, Balneaves LG. A Pap test screening clinic in a South Asian community of Vancouver, British Columbia: challenges to maintaining utilization. *Public Health Nurs* 2004;21(5):412-8.
67. Kinnaird V, Momsen J. Different Places, Different Voices: Gender and Development in Africa, Asia and Latin America. London and New York: Routledge 2002
68. Senarath U, Gunawardena N. Women's Autonomy in Decision Making for Health Care in South Asia. *Asia Pac J Public Health* 2009;21(2):137-43.
69. DH. NHS Cancer Screening, 2015
70. Jain A, Serevitch J. Breast cancer screening - how do we communicate with women of South Asian origin? . *Breast Cancer Research* 2009 11(Suppl 2):1. doi: https://doi.org/10.1186/bcr2400

For peer review only

For peer review only - http://bmjopen.bmj.com/site/about/guidelines.xhtml

Table 1: Inductive studies				
**Region**	**Sample size**	**Sampling frame**	**Focus**	**Findings**
**Breast cancer**				
Ahmad et al, (2012)				
Toronto Canada	60 Indian and Pakistani immigrant women, 50+ years; never screened or screened >3 years ago	Concept mapping. Clustering of participant-generated statements.	Experiences and beliefs concerning barriers to mammography	Barriers to regular screening mammogram: lack of knowledge; fear of cancer and language and transportation. Barriers differed significantly according to years lived in Canada: dependence on family; ease of access to mammogram centre; language and transportation; fear of cancer and self-care.
Bottorff et al (1998)				
Large urban setting, Western Canada	50 SA women, 30+ years. FGDs with 30 mostly new informants.	IDIs and FGDs with healthy immigrant SA women via SA investigators' Networks	Beliefs attitudes and values related to breast health practices and screening	Beliefs centred on 4 domains:1) A woman's calling - keeping the family honour, modesty and putting others first; 2) Beliefs about cancer.; 3) Taking care of your breasts; 4) Accessing services.
Meana et al (2001)				
Toronto, Canada	30 recently immigrated Tamil women from Sri Lanka ≥ 50 years	Members of a SA Women's Centre. 3 FGDs	Attitudes/ beliefs regarding BC and BC screening	Common barriers to BC screening: 1) lack of understanding of the role of early detection in medical care; 2) Religious beliefs; 3) Fear of social stigmatisation. Other barriers: embarrassment about mammography procedures. No reported opposition from husbands.

For peer review only - http://bmjopen.bmj.com/site/about/guidelines.xhtml

Pons-Vigués, et al. (2012a)				
Barcelona, Spain	68 healthy women (6 Pakistani, Indian women), 40-69 years	Key informants, cultural mediators and associations.	Concept of health prevention and knowledge, perceived benefits/ barriers	Health prevention concept lay across 3 axes. 1) *Understanding of prevention*; 2) *Proactive or deterministic conception of health disease*: *3)* women cared little for their own health but obliged to others.
**Cervical cancer**				
Bottorff et al. (2001)				
Western Canada	20 SA (Sikh, Hindu, Muslim) women; 20+ years, had Pap test.	IGIs with SA women attending for Pap testing organised by ethnic group	Experiences and views concerning testing, their expectations and preferences	Perceptions of Pap testing: uncertainty about benefits of early detection in the absence of symptoms; reservations about screening unmarried young women due to preserving virginity; seen as beneficial to keep healthy and protect families from disease. Interplay between cultural values and healthcare system structures: shyness and discomfort discussing Pap test with physician .
Haworth et al (2014a)				
Nebraska mid-western USA	27 healthy Bhutanese refugee women; 19 - 60 years.	Snowball sample community venues and residences (2 FGDs)	CC and screening knowledge; susceptibility severity of CC; benefits/ barriers to screening.	Most women had never heard of CC (or HPV) and felt it did not occur in their community. Women not familiar with concept of health prevention. Barriers: shyness; feelings of exposure and potential stigma; historical abuse, sexual assault and inappropriate behaviour by male HCPs in refugee camps; language; navigating a complex health system; limited insurance coverage; transportation; male translators.
Oelke & Vollman (2007)				

For peer review only - http://bmjopen.bmj.com/site/about/guidelines.xhtml

Urban Canada	53 immigrant Sikh women, 21 to 65+ years. Residency in Canada 6 months - 32 years	Community locations, key contacts and Punjabi radio (13 IDIs). Community agency and English classes (3 FGDs)	Knowledge, understanding and perceptions of CC screening	*'Inside/outside'*: difficult to move 'outside' into Canadian society. *Individual*: unaware of importance of prevention; cervix as unknown body part; SRH not discussed. *Knowledge:* minimal knowledge of Pap test and no ready access to information. *Prevention*: not necessary in absence of symptoms. *Family:* cultural constraints; domination by males/elders; needing permission for medical appointments; a woman's sacrifice for the family. *Community:* preserving honour/status; shame surrounding inappropriate topic. *Healthcare system:* sex of physician; language barriers; trust; confidentiality and dearth of acceptable HCPs.

KEY for all tables:

SA = South Asian
IDI = In-depth Interviews
FGD = Focus Group Discussions
HCP = Healthcare provider or professional
SRH = Sexual and reproductive health
BC = Breast Cancer
CC = Cervical Cancer

For peer review only

For peer review only - http://bmjopen.bmj.com/site/about/guidelines.xhtml

**Table 2 Predictive studies**						
**Region**	**Sample Size**	**Sampling Frame**	**Focus**	**Outcome Variable**	**Rate**	**Risk Factors for not screening**
**Breast Cancer Study**						
Ahmed & Stewart, 2004						
Canada	Cross-section 54 SA women, aged 18+, Hindu or Urdu speakers	Attendees family medical clinic	HBM	Ever had CBE	38.5%	Younger age, more barriers
Boxwala, et al. 2010						
Detroit, USA	Cross-section 160 Indian women, 39+ years	Cultural or religious locations	HBM	Mammogram and CBE within 2 years	63.8%	Not graduate education, disagree screening useful, mammogram less relatively important, not recommended by health professional
Chawla, et al. 2015						
California, USA	Cross-section 186 SA women aged	Random digit	None	Mammography	79.5	Not married, <25% of lifetime in USA, no

For peer review only - http://bmjopen.bmj.com/site/about/guidelines.xhtml

	50 to 74	telephone survey		test within 2 years		physician visits in last year
Hasnain, et al. 2014						
Chicago, USA	Cross-section 105 SA first generation Muslim women	Snowball sample	Anderson Behavioural Model for Health Service Use Anderson Model), HBM, Transtheoretical Model	Mammography test within 2 years (adherent), Mammography test not within 2 years (overdue), never screened	41% Adherent,	Fewer years in the USA, lower mammogram importance, more barriers, lower intention
Islam, et al. 2006						
New York, USA	Cross-section 43 women 18+	Attendees at cultural events	None	Mammogram test within 2 years	55.8%	Uninsured, greater than ten years living in USA
Kwok, et al 2015						
Sydney, Australia	Cross-section 242 women 18+ born in India or in Indian communities	Attendees at cultural events	Culture-specific factors	Have biannual mammography	17.8%	Less time in Australia, divorced, separated, widowed
Marfani, et al. 2013						
Baltimore, USA	Cross-section 418	Attendees at cultural and	Acculturation	Mammography or CBE within 1	Not	Low self and outcome efficacy for screening,

For peer review only

For peer review only - http://bmjopen.bmj.com/site/about/guidelines.xhtml

	Indian women	religious events		year, Mammography or CBE within 2 years, mammography or CBE more than 2 years ago	provided	greater barriers, lower acculturation, lower acculturation interacting with greater anxiety about BC
Meana, et al. 2001						
Canada	Cross-section 122 Tamil women, 50+ years	Attendees at community and religious centres	HBM	Had mammograms	57.4%	Fewer years in North America, More barriers
Menon, et al. 2012						
Chicago, USA	Cross-section 330 SA women 40+ years	Community-based agencies	Precede-Proceed Model	Ever had mammography	65.5%	Less than 5 years in USA, greater barriers, lower English language preference, never had cervical screening
Misra, et al. 2011a						
USA cities	Cross-section 389 Indian women 40+	Random survey	None	Ever had mammography	81.2%	Fewer years in USA, No health insurance
Misra, et al. 2011b						

For peer review only - http://bmjopen.bmj.com/site/about/guidelines.xhtml

USA cities	Cross-section 519 Indian women 18+	Random survey	None	Ever had Pap test	74.2%	Fewer years in USA, Lower education, No health insurance, No family cancer history
Pourat, et al., 2010						
California, USA	Cross-section 134 SA women 40+	Random survey	Acculturation	Mammogram within 2 years	39%	None
Vahabi, et al. (2016)						
Ontario, Canada	18,880 women 50-69	Government. Database linkage study	None	Verified mammography attendance within 2 years	63.7%	Fewer years in Canada, no general GP assessment, GP trained overseas
**Cervical Screen Study**						
Chaudhry, et al. 2003						
USA	Cross-section 225 SA women aged 15-83	SA Family Names	Anderson Model	Pap test within 3 years	73%	Unmarried, not bachelor degree, no usual source of medical care, <25% of lifetime in USA
Chawla, et al. 2015						
California, USA	Cross-section 711 SA women aged	Random digit	None	Pap test within 3	79.5	Younger age, not married,

For peer review only - http://bmjopen.bmj.com/site/about/guidelines.xhtml

	21 to 74	telephone survey		years		<25% of lifetime in USA
Gupta, et al., 2002						
Toronto, Canada	Cross-section 62 SA university students, 62 Tamil women 18-60	Common areas of university, Tamil community centres	Acculturation	Ever had Pap test	25%	Lower education, education outside Canada, lower acculturation
Islam, et al. 2006						
New York, USA	Cross-section 98 women 18+	Attendees at cultural events	None	Pap test within 3 years	54.4%	Tested within 3 years: Lower education, Lower income, Uninsured, less than ten years living in USA
Lin, et al., 2009						
California, USA	Cross-section 338 SA women 18-65	Random telephone survey	None	Pap test in last 3 years	73%	Not married, low income, no usual source of medical care
Menon, et al. 2012						
Chicago, USA	Cross-section 330 SA women 40+ years	Community-based agencies	Precede-Proceed Model	Ever had cervical screen	32.8%	Lower education, greater barriers, lower English language preference, never had mammogram

For peer review only - http://bmjopen.bmj.com/site/about/guidelines.xhtml

Misra, et al. 2011						
USA cities	Cross-section 519 Indian women 18+	Random survey	None	Ever had Pap test	74.2%	Fewer years in USA, Lower education, No health insurance, No family cancer history
Pourat, et al., 2010						
California, USA	Cross-section 195 SA women 40+	Random survey	Acculturation	Pap test within 3 years	73%	Greater distance to Asian clinic, No health insurance, No private doctor, Has previously delayed obtaining medical care, Has had problem obtaining satisfactory doctor over past year

For peer review only

Table 3: Comparative studies				
**Region**	**Sample size**	**Sampling frame**	**Focus**	**Findings**
**Breast cancer**				
Abdul Hadi, et al. (2010)				
Penang State, Malaysia	65 healthy Indian women aged >15, 177 Malay and 121 Chinese	Two shopping malls	Differences in knowledge/perception of breast cancer	Indians less knowledge risk factors, symptoms and screening options (subsidised mammography and CBE) compared to Malay and Chinese. Univariate analysis confounded by Indian population being least educated.
Pons-Vigués, et al. (2012b)				
Barcelona city, Spain	25 Pakistani-Indian women 45-69 years, 275 Spanish women 660 other immigrant groups	Sampled from Census respondents.	Adapted HBM based on qualitative pilot study (Pons-Vigues, et al, 2012a).	Indian-Pakistani women perceived more barriers to mammography screening than host country women, but fewer than other immigrant groups.
Sim et al. (2009)				
Singapore	80 Indian women, 182 Malay, 700 Chinese, 38 other	Visitors to general hospital (not patients)	Knowledge and beliefs about BCa and screening practices	No differences between Indian women and others in either knowledge or having ever attended a screening mammogram.
Teo et al. (2013)				
Singapore	52 locally raised Indian women<br>104 Chinese<br>52 Malay	Female patients and visitors to polyclinic, aged 40-70.	No theoretical model	Indian women less likely to have ever had mammogram compared with majority Chinese, but more likely than Malays. Indian women *least* likely group to cite cost or

For peer review only - http://bmjopen.bmj.com/site/about/guidelines.xhtml

				potential pain as barriers to attending mammography
Vahabi, et al. (2016)				
Ontario, Canada	18,880 South Asian, 85,872 other immigrant groups	Government database linkage study	No theoretical model	Lower mammography attendance in previous 2 years than other immigrant groups
Wu et al. (2006a)				
Michigan, USA	38 Indian women aged ≥40<br>X Chinese<br>X Filipino	Community or religious groups; ethnic student associations, community events	HBM.	No difference in CBE and mammography take up between ethnic groups. Indian women had lower scores on perceived susceptibility and seriousness than Filipino and Chinese controlling income. Indian women more likely to say 'do not know where to find mammogram'.
Wu et al. (2008b)				
Michigan, USA	109 Asian Indians aged ≥40 years, literate	Community events, cultural centres, faith-based organizations, Asian health fairs	HBM.	No group differences.
**Cervical cancer**				
Dunn & Tan (2010)				

For peer review only - http://bmjopen.bmj.com/site/about/guidelines.xhtml

Malaysia	96 married Indian women aged 25-65	2-stage stratified-cluster random sampling	No theoretical model	Ever had Pap test: Indian population least likely to have ever had screening. Indian women who had ever received screening less likely to know its purpose than Malays. Indian women who had never had Pap test were 9% less likely to cite 'embarrassed' as reason for not undergoing testing
Marlow, Wardle and Waller (2015)				
England, UK	120 Indian, 120 Pakistani, 120 Bangladeshi women, 120 white British, 120 Caribbean and 120 African	Quota sampling, random sampling within high ethnic concentration postcodes	No theoretical model	Indian, Pakistani and Bangladeshi women less likely to be screened over last 5 years than white British. Less knowledge than white British

For peer review only - http://bmjopen.bmj.com/site/about/guidelines.xhtml

Table 4: Intervention studies				
**Region**	**Sample Size and Sampling frame**	**Intervention**	**Focus**	**Findings**
**Breast cancer**				
Ahmad et al, 2005				
Toronto, Canada	127 SA immigrant women. Mean age = 37 years (SD 9.7); lived 6 years in Canada (SD 6.6). n=82 pre-intervention; n=74 post intervention	Pre (PrI)- Post (PoI) intervention comprising written socioculturally tailored language-specific health education materials.	Barriers to mammography screening<br><br>HBM ,Stages of Change model)	A significant increase in self-reporting 'ever had' routine physical check-up (46.4-70.8%; $p < 0.01$) and CBE (33.3-59.7%; $p < 0.001$). Decrease in: misperception of low susceptibility to breast cancer women (3.0-2.4; $p < 0.001$); misperception of short survival after diagnosis (2.7-1.8; $p < 0.001$); and perceived barriers to CBE (2.5-2.1; $p < 0.001$). Self-efficacy to have CBE increased (3.1-3.6; $p < 0.001$).
Hoare et al, 1994				
Oldham, UK	5277 women with SA names from general practices with high number of SA patients. Pakistani/intervention n=145 (59%); Bangladeshi/controls n=87 (57%).	RCT: 527 women stratified into Pakistani (n=324) intervention and Bangladeshi (n=203) control groups	Awareness of screening	No difference in attendance was found between the intervention and control groups (49%and 47%). Attendance for screening was related to length of stay in the United Kingdom.
Sadler et al, 2003				

For peer review only - http://bmjopen.bmj.com/site/about/guidelines.xhtml

San Diego County, USA	Asian and Pacific Islander women from San Diego County. Indian n=125. Women aged >20 years (screening from 20 onwards)	Pre and post intervention The Asian Grocery Store based cancer intervention program - incorporating an educational program into women's routine shopping activities	Barriers to mammography screening	Shift toward screening uptake for Chinese and Vietnamese American women who were non-adherent at baseline but no change for Asian Indian and Japanese American women at follow up.
**Cervical Cancer**				
Grewal et al, 2004				
Vancouver, Canada	Specialised Pap Test clinic for SA women. 1995-1998; 61 – 107 – 35 new visits in the intervention. Reasons for non-attendance n=74..	Time series of service use. Community initiative led by SA community health nurses in collaboration with influential women in the SA community, local physicians, and health board authorities. Qualitative interviews with 20 women who attended the Pap test clinic	Awareness of screening	Attendance patterns were not maintained although women had positive experiences. Challenges for ongoing success: 1) maintaining the continued involvement of stakeholders in developing long-term strategies to enhance community awareness about cervical cancer; 2) creating mechanisms to strengthen support from physicians in the community; 3) meeting the needs of the underserved within a specialised health service for SA immigrant women.
McAvoy & Raza, 1991				

For peer review only - http://bmjopen.bmj.com/site/about/guidelines.xhtml

Leicester, UK	737 randomly selected asian women; 18-52 years who were not recorded on the central cytology computer as ever having had a cervical smear. N=578 (declined n=159)	Prospective Cohort RCT study (blinded trial)<br>1) visited, shown a video, n=263;<br>2) visited, shown a leaflet and fact sheet, n=219;<br>3) posted a leaflet and fact sheet, n=131;<br>4) not contacted at all, n=124.	Knowledge of early intervention	Only 6 (5%) of those not contacted and 14 (11%) of those sent leaflets had a smear test during the study. Health education interventions increased the uptake of cervical cytology among women in Leicester who had never been tested. Visits and videos were most effective.
**Breast and cervical cancer**				
Kernohan 1996				
Bradford UK	October 1991 to March 1993, a stratified sample of 1,000 women (670 SA, 163 African-Caribbean, 96 Eastern European and 71 other)	Community development approach - Pre - Post intervention<br>Two Health Promotion Facilitators undertook community development work in both formal and informal settings. Women were interviewed at the beginning of the project and six months after the health promotion intervention.	Knowledge about cervical cancer and breast cancer	SA women had the lowest levels of knowledge and also showed the most significant improvements. Significant increases in attendance for cervical smear and breast cancer screening were self-reported.

For peer review only - http://bmjopen.bmj.com/site/about/guidelines.xhtml

Figure 1: PRISMA Flowchart of the study selection process. Adapted from: PLoS Medicine (OPEN ACCESS) Moher D, Liberati A, Tetzlaff J, Altman DG, The PRISMA Group (2009). Preferred Reporting Items for Systematic Reviews and Meta-Analyses: The PRISMA Statement. PLoS Med 6(7): e1000097. doi:10.1371/journal.pmed1000097.

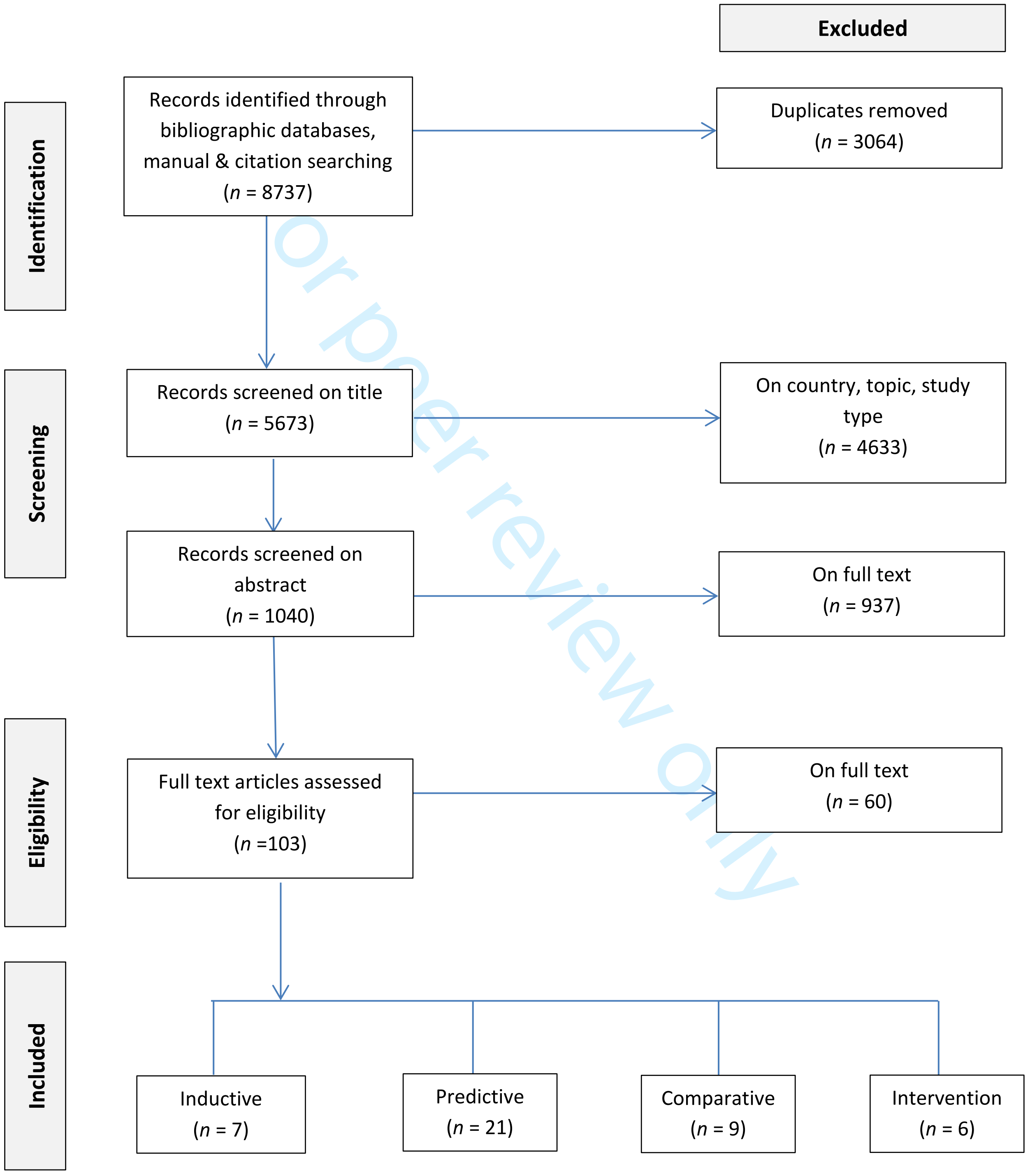

For peer review only - http://bmjopen.bmj.com/site/about/guidelines.xhtml

BMJ Open

A systematic review of barriers and enablers to South Asian women's attendance for asymptomatic screening of breast and cervical cancers in emigrant countries

Journal:	*BMJ Open*
Manuscript ID	bmjopen-2017-020892.R1
Article Type:	Research
Date Submitted by the Author:	08-Mar-2018
Complete List of Authors:	Anderson de Cuevas, Rachel; University of Liverpool, Public Health & Policy<br>Saini, Pooja; Liverpool John Moores University, School of Natural Sciences and Psychology<br>Roberts, Deborah; Royal Liverpool and Broadgreen University Hospitals NHS Trust<br>Beaver, Kinta; University of Central Lancashire; University of Central Lancashire<br>Chandrashekar, Mysore; Royal Liverpool and Broadgreen University Hospitals NHS Trust<br>Jain, Anil; University Hospital of South Manchester NHS Foundation Trust<br>Kotas, Eleanor; University of Liverpool, Health Services Research<br>Tahir, Naheed; University of Liverpool , Health Services Research<br>Ahmed, Saiqa; University of Liverpool, Health Services Research<br>Brown, Stephen; University of Liverpool, Psychological Sciences
**Primary Subject Heading**:	Oncology
Secondary Subject Heading:	Research methods, Public health, Health services research, Evidence based practice
Keywords:	South Asian women, asymptomatic screening, breast cancer, cervical cancer, mammography, smear

SCHOLARONE™
Manuscripts

A systematic review of barriers and enablers to South Asian women's attendance for asymptomatic screening of breast and cervical cancers in emigrant countries

Correspondence: Pooja Saini, School of Natural Sciences and Psychology, Tom Reilly Building, Byrom Street, Liverpool John Moores University, Liverpool, L3 3AF, UK.
Email: P.Saini@ljmu.ac.uk

Rachel Anderson de Cuevas*, University of Liverpool, Whelan Building, Brownlow Hill, University of Liverpool, L69 3GS, UK

Pooja Saini*, NIHR Collaboration for Leadership in Applied Health Research and Care North West Coast and School of Natural Sciences and Psychology, Tom Reilly Building, Byrom Street, Liverpool John Moores University, Liverpool, L3 3AF, UK

*Joint first authorship. Pooja Saini and Rachel Anderson de Cuevas contributed equally.

Deborah Roberts, Royal Liverpool and Broadgreen Hospital NHS Trust, Prescot St, Liverpool, L7 8XP, UK

Kinta Beaver, University of Central Lancashire, School of Health Sciences, Brook Building, Preston, PR1 2HE, UK

Mysore Chandrashekar, Royal Liverpool and Broadgreen Hospital NHS Trust, Prescot St, Liverpool, L7 8XP, UK

Anil Jain, The Nightingale Centre and Genesis Prevention Centre, University Hospital of South Manchester NHS Foundation Trust, Southmoor Rd, Wythenshawe, Manchester, M23 9QZ, UK

Eleanor Kotas, University of Liverpool, Whelan Building, Brownlow Hill, University of Liverpool, L69 3GS, UK

Naheed Tahir, NIHR Collaboration for Leadership in Applied Health Research and Care North West Coast Public Advisor, University of Liverpool, Liverpool, UK

Saiqa Ahmed, NIHR Collaboration for Leadership in Applied Health Research and Care North West Coast Public Advisor, Liverpool, UK

Stephen L. Brown, University of Liverpool, Whelan Building, Brownlow Hill, University of Liverpool, L69 3GS, UK

Word count: 4668 words

For peer review only - http://bmjopen.bmj.com/site/about/guidelines.xhtml

Abstract

Objectives: The aim of this review was to identify the cultural, social, structural and behavioural factors that influence asymptomatic breast and cervical cancer screening attendance in South Asian populations, in order to improve uptake and propose priorities for further research.

Design: A systematic review of the literature for inductive, comparative, prospective and intervention studies. We searched the following databases: MEDLINE/In-Process; Web of Science; EMBASE; SCOPUS; CENTRAL; CDSR; CINAHL; PsycINFO and PsycARTICLES from database inception to 23 January 2018. The review included studies on the cultural, social, structural and behavioural factors that influence asymptomatic breast and cervical cancer screening attendance and cervical smear testing (Papanicolaou test) in South Asian populations and those published in the English language. The Framework Analytic method was used and themes were drawn out following the Thematic Analysis method.

Settings: Asymptomatic breast or cervical screening

Participants: South Asian women, including Bangladeshi, Indian, Pakistani, Sri Lankan, Bhutanese, Maldivian and Nepali populations.

Results: 51 included studies were published between 1991 and 2018. Sample sizes ranged from 25 to 38,733 and participants had a mean age of 18 to 83 years. Our review showed that South Asian women generally had lower screening rates than host country women. South Asian women had poorer knowledge of cancer and cancer prevention and experienced more barriers to screening. Cultural practices and assumptions influenced understandings of cancer and prevention, emphasising the importance of host country cultures and healthcare systems.

For peer review only - http://bmjopen.bmj.com/site/about/guidelines.xhtml

Conclusions: High quality research on screening attendance is required using prospective designs, where objectively-validated attendance is predicted from cultural understandings, beliefs, norms and practices; thus informing policy on targeting relevant public health messages to the South Asian communities about screening for cancer.

Systematic Review Registration: PROSPERO CSD 42015025284

For peer review only

Strengths and limitations of this study

- Separate outcomes were compared of integrative reviews of inductive, predictive, comparative and intervention studies to assess consistencies between methods.
- Inductive studies provided nuanced and detailed insights into cultural, social, structural and behavioural factors influencing screening attendance.
- Deductive studies did not use insights gained from inductive research, were either atheoretical or used generic health psychology theories that were validated on western samples and were generally poorly designed.
- Due to the small number of published studies, it is difficult to identify factors unique to groups of South Asian women based on nationality, geographical region or religion.
- We provide specific advice for high quality deductive research on screening attendance that will allow estimation of the prevalence of factors that facilitate or inhibit screening attendance and the magnitude of their influence on attendance.

For peer review only - http://bmjopen.bmj.com/site/about/guidelines.xhtml

Introduction

Since 1945, many countries have benefited economically and socially from large-scale migration from the South Asian nations of India, Pakistan, Bangladesh, Sri Lanka, Nepal, Maldives and Bhutan [1]. Migration has largely favoured English-speaking countries, although large South Asian populations also exist in non-Anglophone European, African and neighbouring Asian countries [2]. In the United Kingdom (UK), the South Asian population constitutes the largest ethnic minority category [3]. In all host countries, historic migration patterns have led to the establishment of South Asian communities in cities and large towns where cultural norms and practices of the countries of origin are practised alongside those of the host country [4].

In the UK, South Asian women have higher breast and cervical cancer mortality than the host population, worse cancer-related health outcomes, with the exception of some Indian groups, and are more likely to present with advanced disease [3 5]. Whilst South Asian and host populations may differ over a range of factors that influence mortality, such as tumour sub-type and HPV status (Gomez 2010), one potential cause of greater mortality is that South Asian women show a lower likelihood of attending routine mammographic and Papanicolaou (Pap) screening. Screening is widely available in most high income countries [6-8]. Some research shows shows population mortality benefits of screening programmes [10 11], although other studies find no effect [9]. Importantly, greater mortality benefits are found at the individual level, where studies confine analyses to women who accept screening rather than those who are merely invited (because some women decline screening) [12]. Compared with the host population, South Asian women in England show lower uptake of breast screening services [13-16], particularly those from lower socioeconomic groups [13 17 18] and a higher proportion have never received cervical screening [19]. This is also the case in the USA [20].

For peer review only - http://bmjopen.bmj.com/site/about/guidelines.xhtml

Possible explanations for why screening rates are lower in South Asian populations have included poorer individual knowledge and awareness of breast and cervical cancer [21-23], lower community awareness, poor communication between health professionals and patients, health professional background, and less access to appropriate cancer health services [24 25]. Some South Asian women cannot speak or read in the host language [26 27]. Another body of research focuses on South Asian women's attitudes, beliefs and behaviours relating to cancer screening [28 29]. Crawford et al. [29] and Sokal [26] recently compiled scoping and critical reviews of breast, cervical and colorectal screening in South Asian populations in Canada, the USA and the UK. The reviews demonstrate how individuals' beliefs, knowledge and perceptions of access barriers are shaped by the host environment, migration experience, cultural references and practices of the country of origin, and the cultural processes of adaptation to the host country.

Crawford et al. and Sokal's reviews have limitations. Both, combined studies with differing methodological approaches to achieve integrated descriptions of findings. This approach provides a comprehensive overview, but may lead to interpretation bias because it does not separate content from method [26]. This leads to two limitations. First, critical examination of study quality is more difficult when varying methodologies are used. Thus, the value of findings cannot be easily moderated or weighted by quality appraisal of the reviewed studies. Second, it is important that findings are replicated across methods. For example, inductive research permits detailed phenomenological understandings of factors that facilitate or inhibit screening, but not epidemiological estimates of the prevalence of these factors or the magnitude of their influence on screening. This requires well-designed quantitative studies[30]. Similarly, quantitative research alone is unlikely to be sensitive to local complexities unless complemented by inductive approaches. When these approaches are

For peer review only - http://bmjopen.bmj.com/site/about/guidelines.xhtml

conflated, as with Crawford et al. and Sokal's approach, the reader cannot determine if insights are or are not replicated across different approaches.

Whittemore and Knafl [31] describe a method of integrative review that resolves these problems by separately integrating findings within different methodologies, then comparing the integrative findings across analyses to identify consistencies and limitations of findings within and between methods. Researchers into migrant health use four basic types of investigation. *Inductive* studies use qualitative analyses that allow participants to present their own experiences, thus providing novel insights that drive theory development. *Predictive, comparative* and *intervention* studies are deductive, using quantitative methods to test hypotheses. *Predictive* studies predict health behaviour from measures of individual and contextual attributes, allowing theory testing by quantifying associations between predictors and outcomes within migrant populations. *Comparative* studies compare target populations with host or other immigrant populations to identify whether the determinants of health behaviour in immigrant groups are unique to them or are shared with host or other immigrant groups. Shared factors include relative economic deprivation [26 32] or social and cultural adjustment challenges [33]. It is also important to review reports of *intervention* studies to examine how successful previous interventions (or their individual components) have been in improving screening rates in South Asian populations.

Aims of the Review

We examined cultural, social, structural and behavioural factors that influence asymptomatic breast and cervical cancer screening attendance in South Asian populations, to explain why attendance rates are lower than host country women. We performed separate integrative reviews of inductive, predictive, comparative and intervention studies, and compared outcomes of these reviews to assess consistencies between methods. Our aim was to identify

For peer review only - http://bmjopen.bmj.com/site/about/guidelines.xhtml

the cultural, social, structural and behavioural factors that influence asymptomatic breast and cervical cancer screening rates in South Asian populations to improve screening rates and to propose priorities for further research. Our objectives were to:

- critically review and integrate findings of inductive, predictive, comparative and intervention studies on asymptomatic screening;
- document consistent and inconsistent findings across methods; make theoretical and methodological recommendations for the conduct of future research.

For peer review only

For peer review only - http://bmjopen.bmj.com/site/about/guidelines.xhtml

Methodology

Search strategy

We conducted literature searches using multiple databases to overcome problems associated with inadequate indexing [31 34] and to ensure a more exhaustive scope [31 35 36]. We searched the following databases: MEDLINE/In-Process; Web of Science; EMBASE; SCOPUS; CENTRAL; CDSR; CINAHL; PsycINFO and PsycARTICLES for 4 key concepts: 1) South Asian population 2) cancer 3) asymptomatic breast or cervical screening and 4) knowledge, attitude, practice, behaviour or compliance. PubMed was searched for publications ahead of print and conference proceedings. Search terms were revised after initial searches revealed new terms. MeSH terms were run in combination with free-text searches of titles and abstracts. These are available as an online data supplement at https://www.crd.york.ac.uk/PROSPEROFILES/25284_STRATEGY_20170702.pdf. Searches were conducted from database inception to 23 January 2018. The search was restricted to original research in English for all publication dates. Citations of selected studies were reviewed to identify any additional studies. We checked for grey literature via databases and repositories such as Open SIGL, Open Grey, PsycEXTRA, HMIC UK, The Grey Literature Report, ClinicalTrials.gov, NTIS, NCIN and the WHO ICTRP, and cancer and clinical networks including American Cancer Society, South Asian Health Foundation and MacMillan Cancer Support.

Selection criteria

The review included studies on the cultural, social, structural and behavioural factors that influence asymptomatic breast and cervical cancer screening attendance and cervical smear testing (Papanicolaou test) in South Asian populations. It was confined to host countries where mass screening programmes are available to the general public, including South Asian

For peer review only - http://bmjopen.bmj.com/site/about/guidelines.xhtml

Women. The populations of interest were Bangladeshi, Indian, Pakistani, Sri Lankan, Bhutanese, Maldivian and Nepali populations (or ethnic subgroups thereof). To ensure that content was not confounded by inclusion of other groups, studies needed to report on samples or subsamples identifiable as wholly South Asian, meaning that we accepted papers that examined South Asian and other samples provided that authors explicitly specified where South Asian content differed from other samples (inductive studies) or where South Asian samples were analysed separately or were specifically identified in moderation analyses (predictive, comparative or intervention studies).

To ensure that the studies pertained to screening attendance, we excluded those that did not specifically refer to screening. Thus, studies solely covering general attitudes to breast or cervical cancer were excluded. The review did not include breast self-examination, diagnostic screening or visual or tactile examinations by healthcare professionals. We excluded studies of women in known high-risk groups who were engaged in monitoring programmes for genetic risk factors, hereditary breast and ovarian cancer syndrome, premenopausal or familial breast cancer. We excluded homogenous samples restricted to particular demographic groups because these are not population representative (e.g. a study of dental students).

Screening

Team members screened titles and abstracts to identify potentially eligible studies and two reviewers independently considered the eligibility of each of the titles and abstracts. Outputs were compared to detect discrepancies and the agreement rate was 90%. Disagreements over selection of abstracts were resolved by consensus between the team. Calibration of the selection criteria was performed after the first 50 and 100 papers and taking a small sample (15%) of reports from grey and unpublished literature. Two reviewers independently assessed

For peer review only - http://bmjopen.bmj.com/site/about/guidelines.xhtml

For peer review only

the full text of relevant studies using a standardized, pilot-tested screening form agreed with the steering group. Disagreements were resolved by consensus or by referral to a third-party arbiter. EndNote (X5) reference manager was used to manage citations and view abstracts and full-text articles.

Quality evaluation

Each study was evaluated for quality specific to the method used, with validated checklists developed from the Critical Appraisal Skills Programme [37]. Inductive studies were generally found to be good. Predictive, descriptive and intervention studies had theoretical, sampling, design and measurement limitations. We did not exclude studies that used poor methodologies, but extensively describe these problems and consequent interpretive limitations in the results.

Data extraction, synthesis and analysis

All studies included in the review are included in summary tables (Tables 1 to 4). Four reviewers completed data extraction for each study type and reviewed the variable headings upon completion [38]. Subsequently, tables were adapted and the following variables were recorded for all studies: region, study design (sample size and sampling); demographic and clinical characteristics of women selected; setting; data collection instruments; analytic method; nature of asymptomatic screening (mammogram or Pap smear test); definition of timely screening attendance; theoretical focus; key findings; study limitations and quality rating. For predictive studies we recorded outcome variables, rate of screening attendance and all predictors for and against screening. Intervention studies included a description of the intervention concerned.

For peer review only - http://bmjopen.bmj.com/site/about/guidelines.xhtml
For peer review only

Syntheses were made using thematic analysis within each methodology type [31 39]. Syntheses were initially structured from the summary tables, beginning with a period of data familiarisation, during which researchers listed ideas about emerging themes which formed the basis of a thematic framework. At this point, the analysis returned to the full papers, where the developing thematic framework was tested and refined against the initial data. Themes were developed, reviewed and refined by analysing the data synthesised within each code and testing for 'internal homogeneity' and 'external heterogeneity' [40]. The research group met continuously to check and discuss the meaning and interpretation of the data.

Patient and Public Involvement

The research question was derived following the author (PS) attending community intervention sessions with South Asian women. There, the lack of knowledge of female cancers and the stigma associated with female cancer became apparent. Following some discussions, the group were asked about their own experiences and whether they would like to be part of future research to gain more understanding of the cultural, social, structural and behavioural factors that influence breast and cervical cancer screening attendance in South Asian populations. The group of attendees at the community sessions were invited to be involved in a funding application being submitted to the NIHR Collaboration for Leadership in Applied Health Research and Care North West Coast [CLAHRC NWC] and then the research group if the funding was awarded. Two women from the community (NT and SA) were interested then invited to join the research team made up of academics and clinicians. They were then signed up as NIHR CLAHRC NWC Public Advisors. All team members were involved in reviewing the submitted grant application and subsequently attended all steering group meetings where the search terms were finalised for the systematic review. The researchers, a seconded nurse from the local hospital and public advisors attended all training

For peer review only - http://bmjopen.bmj.com/site/about/guidelines.xhtml

associated with conducting a systematic review, reviewed titles, abstracts and full papers for inclusion and exclusion and attended data analysis meetings. The public advisors and main researcher have disseminated the preliminary study findings at national and regional conferences, national meetings, community public engagement events and at the University of Liverpool. Both public advisors have become active members of the wider NIHR CLAHRC NWC structure since joining this review project and other women from the same community are now involved in other studies across the area. Their contribution has been invaluable.

For peer review only

For peer review only - http://bmjopen.bmj.com/site/about/guidelines.xhtml

Results

The combined search of electronic bibliographic databases yielded 10,969 citations (Figure 1: PRISMA Flowchart of the study selection process. Adapted from: Moher, Liberati, Tetzlaff and Altman, 2009). After removing duplicates (n = 3714), the remaining 7255 were screened on title and 1136 on abstract and, 132 records were selected for full-text review. Subsequently, 81 were excluded on full text and 51 met the criteria for inclusion in the review. The 51 studies were published between 1991 and 2017 and were conducted in the USA (n=22), Canada (n=16), UK (n=5), Spain (n=2), Singapore (n=2), Malaysia (n=2), Hong Kong (n=1) and Australia (n=1). Sample sizes ranged from 25 to 38,733. Participants were recruited from community and healthcare settings and had a mean age of 18 to 83 years. Eight were inductive (see Table 1), 25 predictive (containing analysis of predictors of and risk factors for attendance) (see Table 2), 10 comparative (see Table 3), and 8 intervention studies (see Table 4). No further studies were found from the grey literature search.

Overview

Inductive studies provided rich insights into cultural practices and assumptions, and the problems of adjusting to a new social and healthcare system that might inhibit screening in South Asian women. Largely, though, deductive studies failed to exploit these insights in hypothesis testing. Deductive studies were either atheoretical or used generic health psychology theories, such as the health belief model, that were validated on western samples and not adapted for South Asian populations.

Nonetheless, common findings emerged across methodologies. The extent to which women understood the causes of cancer and the benefits of screening were important. Inductive studies revealed cultural constraints on understanding, whilst comparative studies showed South Asian women faring worse on measures of knowledge than host country women. Predictive studies showed that those with more complete understandings of cancer and screening were more likely to attend screening. Similarly, both inductive and deductive studies showed that perceived barriers inhibited screening, and that South Asian women

For peer review only - http://bmjopen.bmj.com/site/about/guidelines.xhtml

typically perceived more and different barriers to host country women. Inductive studies showed the cultural origins of barriers, describing how traditional beliefs about risk, illness, female roles and family structures mitigated screening interest and attendance. Predictive studies showed that the number of perceived barriers inhibited screening, and that South Asian women who were more acculturated to western host countries, operationalized as time spent in those countries, were more likely to attend screening.

Inductive studies

The eight inductive studies (Table 1) were conducted in Canada and the USA, amongst Pakistani, Indian, Sri-Lankan Tamil and Bhutanese populations. Sample sizes ranged from 20-68, with a median of 43. Women had varying lengths of residency and were mostly born outside the host country. Six studies [41-46] used in-depth interviews and/or focus group discussions. One study [47] employed concept mapping using participatory research methods. Pons-Vigues et al. acknowledged difficulties in interviewing women whose cultural backgrounds differed most from their own, and championed the need for cultural intermediaries. However, intermediaries were not used in the other studies. Studies focussed on the experiences of women themselves, and did not interview family members or health care providers.

Data synthesis generated three overarching themes: '*Knowledge, attitudes, understanding of cancer and cancer prevention*; '*Culture*'; and *'The process of Cultural adaptation'* to the host country.

Knowledge, attitudes, understanding of cancer and cancer prevention: Neither cancer nor intimate body parts are commonly discussed in some South Asian cultures [42 46]. All studies

For peer review only - http://bmjopen.bmj.com/site/about/guidelines.xhtml

showed that women lacked basic understandings of cancer, cancer prevention or early detection. Breast cancer was viewed by some women as a '*white woman's disease'* [41], that did not occur in their community [45]. Others considered cancer to be incurable and early detection and intervention futile [45]. Cervical cancer was often not known or understood. For example, some Bhutanese refugees in the USA had not heard of cervical cancer [45]. Indian Sikhs in Canada, living in a culture where sexual and reproductive health is rarely discussed, referred to the cervix as an 'unknown' and unspoken part of their body [46]. Those aware of cervical cancer perceived the principal risk factors to be inseparable from those for general health, rarely mentioning the discrete risk factors of having multiple sexual partners, not using barrier contraception or screening [45].

Studies attributed a lack of understanding of cancer to two main factors. First, religious fatalism meant that cancer was seen as predestined, as divine retribution for sins, or as a dearth of moral character [42]. Second, all studies pointed to the curative focus of healthcare in countries of origin as a reason for some women's failure to understand the concept of prevention [42,48] and consequent belief that healthcare seeking is unnecessary in the absence of symptoms [41,44,46,49].

<u>Culture</u>: Family responsibilities were salient to women. This had three implications, one positive and two negative. First, women felt strong responsibilities to remain in good health and to protect family members from cancer. [40] In some cases this facilitated screening attendance, however, some women found no time to attend screening due to family responsibilities.[44,49] This facilitated screening attendance. Second, notions of stigma precluded screening. Themes of 'shyness', 'modesty' and 'embarrassment' about revealing intimate body parts were important [41]. For example, Bhutanese refugees worried that attending a Pap test would damage reputations for chastity [45]. A Canadian study showed that

For peer review only - http://bmjopen.bmj.com/site/about/guidelines.xhtml

women may be more likely to attend a cervical smear if the family doctor was female [49]. Both breast and cervical cancer were seen as stigmatising [46] and to some women this extended to screening [42 45]. Indeed, some Sri Lankan Tamils worried that attending a mammogram would lead people to think they already had breast cancer [42]. Third, women's behaviour was often subject to influence from male members of the family. Women frequently followed family advice for healthcare provided by males and elders, generally against screening, and felt the need to avoid conflict within the family associated with assertions of independence [41 46]. Another study showed that women felt family members to be supportive [49].

The process of Cultural Adaptation: South Asian nations have largely curative health systems, in which health costs are required to be paid by patients and there is no free access to healthcare. This contrasts with preventive healthcare models in host countries, and as with other health issues, South Asian women showed little understanding or orientation toward cancer prevention [46], although this evolved with time as awareness of the culture of the host country increased [43 47]. Women appreciated healthcare professionals who understood and respected values of personal modesty/shyness [45]. South Asian women in Canada emphasised the value of being chaperoned to screening appointments that may have been located away from their local community, for assistance with language barriers, to alleviate feelings of personal vulnerability and to avoid being alone with doctors [41].

Deductive Studies

Study Quality: Predictive and comparative studies contained similar limitations to quality. The first limitation was the poverty of theory. With the exception of Pons-Vigues and colleagues, whose deductive study [50] was informed by their earlier inductive work [43], we noted little correspondence between inductive themes and hypotheses tested in the deductive

For peer review only - http://bmjopen.bmj.com/site/about/guidelines.xhtml

research. Studies focussed on knowledge of cancer and screening, but were not informed by themes of fatalism, non-understanding of preventive healthcare, or cultural and family systems found in the qualitative research. Instead, studies were theoretically based upon western health behaviour theories, such as the health belief model (HBM) [51], with limited applicability to South Asian populations. Similarly, the concept of acculturation was invoked in predictive studies, but was operationalised in a limited way, focussing on time spent in the emigrant country and language preferences. Other deductive work was not theoretically-based.

Studies were also affected by methodological limitations. Three database linkage studies (from the same research team) [52-54] and two cluster sampling studies [55 56] provided samples with a potentially high degree of population representativeness, with random digit dialling techniques providing some confidence that samples may be representative [57 58]. Other studies used poor sampling techniques, including selection of South Asian names from phone directories or sampling at cultural events or other locations with high proportions of South Asian women, providing less confidence. One comparative study recruited a local population through random-digit dialling, but gathered a convenience sample of SA women through community centres and associations. This difference in sampling means reduces the value of the comparison between samples [59]. Definition of a South Asian population differed between studies, some examined women born in South Asia, others second generation immigrants, and some examined self-identified ethnicity.

It is important that attendance is recorded objectively [60]. All studies but the three linkage studies [52-54] used non-verified self-reported attendance and one used a hypothetical scenario of an offer to attend screening [57]. These outcomes included timely screening attendance (e.g., previous screening was within a specified time period or reported regular timely testing) or whether women had ever been screened in the past.

For peer review only - http://bmjopen.bmj.com/site/about/guidelines.xhtml

Predictive Studies: It is strongly recommended that predictive studies be conducted prospectively to eliminate the problem of reverse causality [60]. All of the 23 predictive studies were cross-sectional and causal interpretation is difficult.

Lower screening rates were noted among women with no health insurance, younger women and women with lower levels of education. Studies did not provide consistent evidence that low knowledge predicted reduced likelihood of attendance. Lower knowledge was associated with a reduced likelihood of mammography screening in two studies [61 62], but did not predict the likelihood of hypothetical acceptance of a cervical screen [57]. Lower attendance was associated with a greater number of self-reported barriers to screening [61-65] although one study found the opposite [66]. However, the instruments used to assess barriers were largely based upon existing instruments developed among western samples that do not reflect South Asian concerns such as adapting to a new culture, language or health system.

Where acculturation was examined, less time spent in the host country was the strongest predictor of non-attendance, although one study cited lower preference for the host language (usually English) compared to women's native language [65] and another self-perceived poorer command of the host language [66]. Vahabi, et al. [52] found that South Asian women were less likely to attend mammography screening if their GP had qualified outside the host country. Lofters, et al. 2017 found that South Asian women were less likely to attend mammography screening if their GP had qualified outside the host country. Vahabi 2017 and Lofters 2017 showed benefits in mammography and cervical screening respectively for female family doctors.

Comparative Studies: Nine of the ten comparative studies compared South Asian women with host populations, eight compared South Asian women to other minority groups. South

For peer review only - http://bmjopen.bmj.com/site/about/guidelines.xhtml

Asian samples often differed from comparison samples on demographic variables such as socioeconomic status (from lower socioeconomic backgrounds) and relationship status (mostly married), which limits trust that can be placed on comparisons if these factors are not statistically adjusted for.

Four comparisons with host populations showed South Asian women to have lower screening rates [55 56 59 67], but two did not [68 69]. Of these studies, Dunn and Tan and Marlow et al. used sampling techniques more likely to derive representative samples. Lower screening rates may be attributable to the knowledge deficits and greater perceived barriers observed in some studies [50 56 70].

Two methodologically rigorous comparisons between South Asian and other minority groups [52 55] used population sampling and statistically adjusted for demographic differences between samples. Vahabi, et al [52] also used an objectively verified indicator of mammography attendance. Both showed South Asian women to have lower attendance rates than other immigrant women. In two studies, Indian women had lower knowledge of cancer and screening than Chinese or Malays [55 70]. Pons-Vigues et al. [50] and Teo et al [67] showed Indian and Pakistani women perceived fewer barriers arising from lack of knowledge about preventative screening than other immigrant groups, and highlighted that many of the women thought that routine blood tests and urine tests would detect broader health issues such as cancer [50]. In another study [68], Indian women perceived themselves to be less vulnerable to getting breast cancer, did not view breast cancer as a serious illness and were more likely to claim that they did not know 'where to find a mammogram'.

<u>Intervention Studies</u>: Community educational programmes promoted breast and cervical cancer screening across the eight intervention studies. Four of the studies were pre-and post-community-based interventions [71-74], two were randomised control trials [48 75]; one a time

For peer review only - http://bmjopen.bmj.com/site/about/guidelines.xhtml

series study [76] and one a snowballing technique used as part of quality improvement initiatives for Physicians [77]. Sampling was predominantly among South Asian women as a group, which eliminates comparisons between the different South Asian populations. Studies employed various methods of socioculturally-tailored, language-specific health education materials and participants were recruited from primary care or South Asian community venues and residences. Recruitment was opportunistic via local newspapers, surveys conducted in community settings, South Asian nurses and link health workers. No study examined age trends [73]; and participants had met the researchers before which may constitute a bias [48]. Controlled studies were conducted in close-knit communities which may have led to intervention contamination into the control groups. Increased screening rates were reported for four studies but many were self-reported [71 72 77] or were indicated to improve [74 77]; rather than from objective indicators [48]. No long-term change in screening uptake was reported for five studies [73-77], but they showed an increase in knowledge of breast cancer among South Asian immigrant women and reduced the misperception of short survival after diagnosis.

For peer review only - http://bmjopen.bmj.com/site/about/guidelines.xhtml

Discussion

Prominent across study types were the findings that South Asian women had poorer understandings of cancer and cancer prevention and that they perceived greater cultural and structural barriers to screening than host country women.

Lack of understanding by South Asian women about the need for asymptomatic screening has important ramifications. Predictive studies showed greater knowledge to be associated with screening attendance. The inductive research yielded some plausible reasons for this. Many women held fatalistic views or beliefs that cancer is incurable, whilst others believed that cancers could be identified in routine health testing. Others were unaware of the existence of cervical cancer in particular, and did not perceive threat to themselves or their communities. The role of males was also important, with male family members sometimes negative about screening and women unwilling to provoke conflict within the family by attending. Whilst there is a clear need to change such beliefs, the inductive studies showed this to be a challenging task for two reasons. First, understandings were embedded within religious and cultural traditions, and cannot be addressed in isolation to those traditions. Thus, a simple educational intervention is likely to have limited effect. Accommodations will need to be reached with communities that allow a creative integration of cancer awareness within existing belief structures. Second, some women were largely unaware of the concept of disease prevention. Thus, the promotion of specific cancer awareness and understandings are unlikely to be helpful until a wider understanding of prevention is reached.

Predictive studies showed the importance of perceived barriers (e.g. lack of education, no health insurance, no family history, lower mammogram importance, less years living in host country, unmarried, language barriers, low self and outcome efficacy for screening), but these barriers pertained only to generic barriers faced by either all women or all immigrant women, irrespective of culture. Acculturation, in terms of time spent in the host country and

For peer review only - http://bmjopen.bmj.com/site/about/guidelines.xhtml

mastery of the language was associated with increased screening likelihood, but these issues are likely to exist for all immigrant women and fail to reveal specifically South Asian issues. Inductive studies provided more subtle and culture-specific indications of the barriers perceived by women. Many were cultural. In particular, women spoke of the importance of female modesty and stigma associated with cancer that also affected willingness to be screened. Whilst the importance of female testing staff from South Asian backgrounds and use of South Asian chaperones is emphasised, this cannot address the wider cultural issues of modesty and stigma. One finding that offers encouragement is that personal health is important to South Asian women because it helps them to care for their families.

Interventions will need to be conducted more widely than merely targeting women and their beliefs. Males occupy decision-making roles in some South Asian families and women may not wish to challenge this (see also [78 79]). Thus, addressing the views of male family members and other community opinion leaders is also important.

Limitations

The following limitations were identified within the review. First, many of the included studies were conducted in the USA, where screening services can require payment, which may not be comparable to other health services. Second, due to the small number of published studies, it is difficult to identify factors unique to groups of South Asian women based on nationality, geographical region or religion. By necessity, we discuss findings in terms of a generic ‘South Asian’ population, but are aware of variance between South Asian populations according to nationality, region, culture and religion. Finally, few studies used sampling techniques that are population representative, employing samples based around community activities. This may introduce unknown biases in findings associated with non-sampling of women who are less likely to attend such activities.

For peer review only - http://bmjopen.bmj.com/site/about/guidelines.xhtml

Future Research

Stratifying the analysis by study methodology brings two benefits; greater confidence can be placed on findings that transcend methodologies than those that are contained within one method, and studies with similar methodologies can be critiqued in ways appropriate to those methodologies. This review emphasises the generally poor quality of the deductive literature, which is problematic for developing epidemiological estimates of the prevalence of factors that inhibit or facilitate screening and the extent to which they do so. Such estimates would provide information pertaining to the relative importance of facilitators and inhibitiors, and how changing them may influence screening attendance[30]. Failure to incorporate inductive findings into the design of deductive studies means that many inductive findings are untested in a population context. Further, deductive studies themselves used flawed designs as they were generally atheoretical or based upon health behaviour models developed in western populations and thus potentially lacking insight into South Asian issues. Translation of inductive findings to a deductive context will require the development of valid and reliable instruments to assess cultural understandings, beliefs, norms and practices.

There is room for well-designed operations research for interventions that target South Asian women who under-utilise and who have never been screened. These studies will also need to use better empirical methods. Few studies used sampling techniques that can be confidently claimed to be population-representative. Thus, there is a risk that South Asian people who attend community events, which was a common sampling strategy, are not representative of those who do not. It is important to employ best practice in study design for screening attendance research; the use of prospective predictive studies and objectively verified reporting of attendance from clinical records [60]. Adequate sampling frames need to be established. Firstly, this involves a distinction between South Asian women as a minority

For peer review only - http://bmjopen.bmj.com/site/about/guidelines.xhtml

group or as an immigrant group. The former can comprise women with high degrees of familiarity with the host country, but who nonetheless may be faced with cultural barriers deriving from their countries of origin. The latter group will reflect the problems of adjustment faced by recent immigrants. Studies will also need to use population-representative sampling techniques.

Recommendations for Practice

Findings from all study types demonstrate that interventions should be sensitive to cultural norms. In particular, studies emphasised the importance of language, female practitioners and the importance of community approval and involvement. Interventions at the community level will be necessary to surmount the cultural barriers identified in the inductive studies.

It is worrying that the findings indicated that younger women and women with lower levels of education were less likely to attend for screening. There is some evidence that South Asian women might experience breast cancer at an earlier age[80], thus interventions may need to be targeted at educating South Asian women who are younger. Encouraging female family members to become more involved as chaperones and translators could also be helpful, and may form a mechanism for educating young women simultaneously. Encouraging female family members to become more involved as chaperones and translators could also be helpful, and may form a mechanism for educating young women simultaneously.

Information aimed at South Asian women who are invited for breast and cervical screening should highlight the presence of female practitioners and exclusively female environments at breast and cervical screening sites in the UK [81]. There is limited use of written communication in South Asian languages, although 70% of screening units across the UK want to provide information in patient’s language [82]. This may help improve South Asian

For peer review only - http://bmjopen.bmj.com/site/about/guidelines.xhtml

women's knowledge, make informed choice/ consent, have better patient experience and eventually help in improving their screening uptake rates.

Interventions to increase uptake rates need to be long-term, multifaceted and tailored to the specific needs of the local community by, for example, developing close links with the community through Health Education workers. South Asian community members, including males and opinion leaders, should be encouraged to be involved and co-produce engagement strategies within community settings. Reducing ethnic inequalities in uptake rates of breast cancer screening needs to remain a policy priority of breast screening programmes.

For peer review only

For peer review only - http://bmjopen.bmj.com/site/about/guidelines.xhtml

Funding

PS and SB are funded by the National Institute for Health Research Collaboration for Leadership in Applied Health Research and Care North West Coast (NIHR CLAHRC NWC) (Grant/Award Number: CLAHRC-NWC-015).

Acknowledgements

The authors thank Professor Andy Clegg, the Royal Liverpool and Broadgreen Hospital Trust, the PREVENT Breast Cancer Charity in Manchester and the South Asian Women who contributed to the study at public engagement events.

Contributions

P.S. & S.B. designed this study; P.S., S.B., R.AdC., D.R., N.T., S.A., K.B., A.J., M.C. and E.K. contributed in finalising search terms; R.AdC., E.K. and D.R. searched databases and R.AdC., D.R and P.S.. collected full-text papers; P.S., S.B., R.AdC. and D.R. extracted and analyzed data; N.T. and S.A. contributed with reviewing abstracts, reading full text papers and extracting data. P.S., R.AdC. and S.B. wrote the manuscript, K.B. reviewed the manuscript. We would like to thank both the public advisers (N.T. and S.A) who contributed throughout this systematic review and which them both luck in any future research.

Competing Interests

The views expressed are those of the author(s) and not necessarily those of the NHS, the NIHR or the Department of Health.

Conflicts of Interest

The Authors declare no conflict of interest.

For peer review only - http://bmjopen.bmj.com/site/about/guidelines.xhtml

Data sharing

No additional data available.

For peer review only

For peer review only - http://bmjopen.bmj.com/site/about/guidelines.xhtml

References

1. ONS. Cancer Registration Statistics , England: 2013: Office for National Statistics, 2013
2. UN. Human Development Reports. United Nations Development Programme, 2008
3. ONS. Cancer Registration Statistics, England: 2011: Office for National Statistics, 2011.
4. Ballard R. The South Asian presence in Britain and its transnational connections. In: Singh H, Vertovec S, eds. Culture and economy in the Indian diaspora. London: Routledge 2002:197-222.
5. Forbes LJ, Atkins L, Thurnham A, et al. Breast cancer awareness and barriers to symptomatic presentation among women from different ethnic groups in East London. *Br J Cancer* 2011;105(10):1474-79.
6. WHO. WHO Guidance Note. Comprehensive cervical cancer prevention and control: A healthier future for girls and women 2013 [Available from: http://apps.who.int/iris/bitstream/10665/78128/3/9789241505147_eng.pdf.
7. WHO. Position Paper on Mammography Screening 2014 [Available from: http://www.who.int/cancer/publications/mammography_screening/en/ accessed 19th August 2015 2015.
8. WHO. Breast Cancer: prevention and control 2015 [Available from: http://www.who.int/cancer/detection/breastcancer/en/ accessed 25 August 2015 2015.
9. Miller AB, Wall C, Baines CJ, et al. Twenty five year follow-up for breast cancer incidence and mortality of the Canadian National Breast Screening Study: randomised screening trial. *BMJ* 2014;348(g366):10. doi: 10.1136/bmj.g366
10. Yip C-H, Smith RA, Anderson BO, et al. Guideline Implementation for Breast Healthcare in Low- and Middle-Income Countries: Early Detection Resource Allocation. *Supplement to Cancer* 2008
11. Marmot MG, Altman DG, Cameron DA, et al. The benefits and harms of breast cancer screening: an independent review - The Independent UK Panel on Breast Cancer Screening. *British Journal of Cancer* 2013;108:2205–40. doi: 10.1038/bjc.2013.177
12. Jacklyn G, Glasziou P, Macaskill P, et al. Meta-analysis of breast cancer mortality benefit and overdiagnosis adjusted for adherence: improving information on the effects of attending screening mammography. *British Journal of Cancer* 2016;114:1269-76. doi: 10.1038/bjc.2016.90
13. Hoare T. Breast screening and ethnic minorities. *British Journal of Cancer* 1996;74(Supplement XXIX):S38 - S41.
14. Waller M, Moss S, Watson J, et al. The effect of mammographic screening and hormone replacement therapy use on breast cancer incidence in England and Wales. *Cancer epidemiology, biomarkers & prevention : a publication of the American Association for Cancer Research, cosponsored by the American Society of Preventive Oncology* 2007;16(11):2257-61. doi: 10.1158/1055-9965.EPI-07-0262 [published Online First: 2007/11/17]
15. Moser K, Patnick J, Beral V. Inequalities in reported use of breast and cervical screening in Great Britain: analysis of cross sectional survey data. *Bmj* 2009;338:b2025. doi: 10.1136/bmj.b2025 [published Online First: 2009/06/18]
16. Robb K, Power E, Atkin W, et al. Ethnic differences in participation in flexible sigmoidoscopy screening in the UK. *Journal of Medical Screening* 2008;15:130-36. doi: 10.1258/jms.2008.007112
17. Edmundson C, Jain A, Astley S. The impact of socioeconomic factors and ethnicity on breast screening uptake in the North West of England. Poster. *British Journal of Radiology* 2009;Supplements
18. Tollitt J, Jain A, Astley S. Health inequalities in breast cancer screening. *Breast Cancer Research* 2008;Suppl 2 (10):1. doi: doi:10.1186/bcr1941

For peer review only - http://bmjopen.bmj.com/site/about/guidelines.xhtml

19. Webb R, Richardson J, Esmail A, et al. Uptake for cervical screening by ethnicity and place-of-birth: a population-based cross-sectional study. *Journal of public health* 2004;26(3):293-6. doi: 10.1093/pubmed/fdh128 [published Online First: 2004/09/30]
20. Shoemaker M, White M, Shoemaker ML, et al. Breast and cervical cancer screening among Asian subgroups in the USA: estimates from the National Health Interview Survey, 2008, 2010, and 2013. *Cancer Causes & Control* 2016;27(6):825-29. doi: 10.1007/s10552-016-0750-5
21. Scanlon K, Wood A. Breast cancer awareness in Britain: are there differences based on ethnicity? *Diversity in Health & Social Care* 2005;2(3)
22. DH. Cancer Reform Strategy: Achieving local implementation - second annual report. NHS. In: DoH, ed.: www.dh.gov.uk/publications, 2009.
23. Johnson C, Mues K, Mayne S, et al. Cervical cancer screening among immigrants and ethnic minorities: a systematic review using the Health Belief Model. *J Low Genit Tract Dis* 2008;12:232-41.
24. Zaman MJS, Mangtani P. Changing disease patterns in South Asians in the UK. *Journal of the Royal Society of Medicine* 2007;100(June):254-55.
25. Elkan R, Avis M, Cox K, et al. The reported views and experiences of cancer service users from minority ethnic groups: a critical review of the literature. *European Journal of Cancer Care* 2007;16:109-21.
26. Sokal R. A critical review of the literature on the uptake of cervical and breast screening in British South Asian women. *Qual Prim Care* 2010;18(4):251-61.
27. Jain A. UK: South Asian Women at the Crossroads NHS Evidence Ethnicity and Health ed, 2010.
28. Wu TY, Guthrie BJ, Bancroft JM. An integrative review on breast cancer screening practice and correlates among Chinese, Korean, Filipino, and Asian Indian American women. *Health Care Women Int* 2005;26(3):225-46.
29. Crawford J, Ahmad F, Beaton D, et al. Cancer screening behaviours among South Asian immigrants in the UK, US and Canada: a scoping study. *Health & social care in the community* 2016;24(2):123-53. doi: 10.1111/hsc.12208 [published Online First: 2015/02/28]
30. Bartholomew LK, Parcel GS, Kok G. Intervention mapplin: A process for developing theory- and evidence-based health education programmes. Health Educ Behav 1998 25(5):545-563.
31. Whittemore R, Knafl K. The integrative review: updated methodology. *Journal of Advanced Nursing* 2005;52(5):546-53.
32. Szczepura A, Price C, Gumber A. Breast and bowel cancer screening uptake patterns over 15 years for UK south Asian ethnic minority populations, corrected for differences in socio-demographic characteristics. *BMC Public Health* 2008;8:346. doi: http://dx.doi.org/10.1186/1471-2458-8-346
33. Marlow L, Wardle J, Waller J. Understanding cervical screening non-attendance among ethnic minority women in England. *British Journal of Cancer* 2015;113:833-39. doi: 10.1038/bjc.2015.248
34. Cooper H. Synthesizing Research: A Guide for Literature Reviews. Second ed. California: Sage Publications 1998.
35. Counsell C. Formulating questions and locating primary studies for inclusion in systematic reviews. *Annals of Internal Medicine* 1997;127:380-87.
36. Jackson N, Waters E. Criteria for the Systematic Review of Health Promotion and Public Health Interventions. *Health Promotion International* 2005;20(4):367-74.
37. CASP. Critical Appraisal Skills Programme (CASP) Checklist Oxford: CASP; 2006 [Available from: www.casp-uk.net/ accessed 1 October 2015.
38. Moher D, Liberati A, Tetzlaff J, et al. Preferred Reporting Items for Systematic Reviews and Meta-Analyses: The PRISMA Statement. *PLoS Med* 2009;6(7) doi: 10.1371/journal.pmed.1000097
39. Braun V, Clarke V. Using thematic analysis in psychology. *Qualitative research in psychology* 2006;3(2):77-101.

For peer review only - http://bmjopen.bmj.com/site/about/guidelines.xhtml

40. Patton MQ. Qualitative evaluation and research methods, 2nd ed. Thousand Oaks, CA, US: Sage Publications, Inc 1990.
41. Bottorff JL, Johnson JL, Bhagat R, et al. Beliefs related to breast health practices: the perceptions of South Asian women living in Canada. *Soc Sci Med* 1998;47(12):2075-85.
42. Meana M, Bunston T, George U, et al. Older immigrant Tamil women and their doctors: attitudes toward breast cancer screening. *J immigr health* 2001;3(1):5-13.
43. Pons-Vigues M, Puigpinos-Riera R, Rodriguez D, et al. Country of origin and prevention of breast cancer: beliefs, knowledge and barriers. *Health Place* 2012;18(6):1270-81. doi: http://dx.doi.org/10.1016/j.healthplace.2012.09.008
44. Bottorff JL, Balneaves LG, Sent L, et al. Cervical cancer screening in ethnocultural groups: case studies in women-centered care. *Women Health* 2001;33(3-4):29-46.
45. Haworth R, Margalit R, Ross C, et al. Knowledge, Attitudes, and Practices for Cervical Cancer Screening Among the Bhutanese Refugee Community in Omaha, Nebraska. *J Community Health* 2014;39(5):872-78 7p. doi: 10.1007/s10900-014-9906-y
46. Oelke ND, Vollman AR. "Inside and outside": Sikh women's perspectives on cervical cancer screening. *Can J Nurs Res* 2007;39(1):174-89.
47. Ahmad F, Mahmood S, Pietkiewicz I, et al. Concept mapping with South Asian immigrant women: barriers to mammography and solutions. *J Immigr Minor Health* 2012;14(2):242-50. doi: http://dx.doi.org/10.1007/s10903-011-9472-7
48. McAvoy BR, Raza R. Can health education increase uptake of cervical smear testing among Asian women? *BMJ (Clinical research ed)* 1991; 302(6780). http://onlinelibrary.wiley.com/o/cochrane/clcentral/articles/153/CN-00075153/frame.html.
49. Hulme J, Moravac C, Ahmad F, et al. "I want to save my life": Conceptions of cervical and breast cancer screening among urban immigrant women of South Asian and Chinese origin. *BMC Public Health* 2016;16(1):1077.
50. Pons-Vigues M, Puigpinos-Riera R, Serral G, et al. Knowledge, attitude and perceptions of breast cancer screening among native and immigrant women in Barcelona, Spain. *Psychooncology* 2012;21(6):618-29. doi: http://dx.doi.org/10.1002/pon.1940
51. Becker M. The Health Belief Model and personal health behavior. *Health Education Monographs* 1974;2:324-508.
52. Vahabi M, Lofters A, Kumar M, et al. Breast cancer screening disparities among urban immigrants: a population-based study in Ontario, Canada. *BMC Public Health* 2015;15:679. doi: http://dx.doi.org/10.1186/s12889-015-2050-5
53. Vahabi M, Lofters A, Kim E, et al. Breast cancer screening utilization among women from Muslim majority countries in Ontario, Canada. *Prev Med* 2017;105:176-83. doi: 10.1016/j.ypmed.2017.09.008

10.1016/j.ypmed.2017.09.008. Epub 2017 Sep 13. [published Online First: 09/17]
54. Lofters AK, Vahabi M, Kim E, et al. Cervical Cancer Screening among Women from Muslim-Majority Countries in Ontario, Canada. *Cancer Epidemiol Biomarkers Prev* 2017;26(10):1493-99. doi: 10.1158/1055-9965.Epi-17-0323

10.1158/1055-9965.EPI-17-0323. Epub 2017 Sep 22. [published Online First: 09/25]
55. Dunn RA, Tan AK. Cervical cancer screening in Malaysia: Are targeted interventions necessary? *Soc Sci Med* 2010;71(6):1089-93. doi: http://dx.doi.org/10.1016/j.socscimed.2010.06.016
56. Marlow LA, Wardle J, Waller J. Understanding cervical screening non-attendance among ethnic minority women in England. *Br J Cancer* 2015;113(5):833-9. doi: http://dx.doi.org/10.1038/bjc.2015.248
57. Chawla N, Breen N, Liu B, et al. Asian American women in California: a pooled analysis of predictors for breast and cervical cancer screening. *Am J Public Health* 2015;105(2):e98-e109. doi: http://dx.doi.org/10.2105/AJPH.2014.302250

For peer review only - http://bmjopen.bmj.com/site/about/guidelines.xhtml

58. Lin MK, Moskowitz JM, Kazinets G, et al. Adherence to pap test guidelines: Variation among Asians in California. *Ethnicity and Disease* 2009;19(4):425-32.
59. So WKW, Wong CL, Chow KM, et al. The uptake of cervical cancer screening among South Asians and the general population in Hong Kong: A comparative study. *Journal of Cancer Policy* 2017;12:90-96. doi: http://dx.doi.org/10.1016/j.jcpo.2017.03.015
60. Sabatino SA, Lawrence B, Elder R, et al. Effectiveness of interventions to increase screening for breast, cervical, and colorectal cancers: nine updated systematic reviews for the guide to community preventive services. *American journal of preventive medicine* 2012;43(1):97-118. doi: 10.1016/j.amepre.2012.04.009 [published Online First: 2012/06/19]
61. Hasnain M, Menon U, Ferrans CE, et al. Breast cancer screening practices among first-generation immigrant muslim women. *J Womens Health (Larchmt)* 2014;23(7):602-12. doi: http://dx.doi.org/10.1089/jwh.2013.4569
62. Marfani F, Rimal RN, Juon H-S. Understanding immigrant women's information needs: Role of acculturation in breast cancer prevention among immigrant Asian Indian women. *J Appl Commun Res* 2013;41(2):126-40. doi: 10.1080/00909882.2012.754485
63. Ahmed NU, Fort JG, Elzey JD, et al. Empowering factors in repeat mammography: Insights from the stories of underserved women. *J Ambulatory Care Manage* 2004;27(4):348-55.
64. Meana M, Bunston T, George U, et al. Influences on breast cancer screening behaviors in Tamil immigrant women 50 years old and over. *Ethn Health* 2001;6(3-4):179-88.
65. Menon U, Szalacha LA, Prabhughate A. Breast and cervical cancer screening among South Asian immigrants in the United States. *Cancer Nurs* 2012;35(4):278-87. doi: http://dx.doi.org/10.1097/NCC.0b013e31822fcab4
66. Kue J, Hanegan H, Tan A. Perceptions of Cervical Cancer Screening, Screening Behavior, and Post-Migration Living Difficulties Among Bhutanese-Nepali Refugee Women in the United States. *J Community Health* 2017;42(6):1079-89. doi: 10.1007/s10900-017-0355-2

10.1007/s10900-017-0355-2. [published Online First: 04/30]
67. Teo CT, Yeo YWS, Lee SC. Screening mammography behavior and barriers in Singaporean Asian women. *Am J Health Behav* 2013;37(5):667-82. doi: 10.5993/AJHB.37.5.11
68. Wu TY, West B, Chen YW, et al. Health beliefs and practices related to breast cancer screening in Filipino, Chinese and Asian-Indian women. *Cancer Detect Prev* 2006;30(1):58-66.
69. Wu TY, Hsieh HF, West BT. Demographics and perceptions of barriers toward breast cancer screening among Asian-American women. *Women Health* 2008;48(3):261-81. doi: http://dx.doi.org/10.1080/03630240802463384
70. Abdul Hadi M, Hassali MA, Shafie AA, et al. Knowledge and perception of breast cancer among women of various ethnic groups in the state of Penang: a cross-sectional survey. *Med Princ Pract* 2010;19(1):61-7. doi: http://dx.doi.org/10.1159/000252837
71. Ahmad F, Cameron JI, Stewart DE. A tailored intervention to promote breast cancer screening among South Asian immigrant women. *Soc Sci Med* 2005;60(3):575-86.
72. Kernohan EE. Evaluation of a pilot study for breast and cervical cancer screening with Bradford's minority ethnic women; a community development approach, 1991-93. *Br J Cancer Suppl* 1996;29:S42-6.
73. Sadler GR, Ryujin L, Nguyen T, et al. Heterogeneity within the Asian American community. *Intern* 2003;2:1-9. doi: http://dx.doi.org/10.1186/1475-9276-2-1
74. Ornelas IJ, Ho K, Jackson JC, et al. Results From a Pilot Video Intervention to Increase Cervical Cancer Screening in Refugee Women. *Health Educ Behav* 2017:1090198117742153. doi: 10.1177/1090198117742153

10.1177/1090198117742153. [published Online First: 12/06]
75. Hoare T, Thomas C, Biggs A, et al. Can the uptake of breast screening by Asian women be increased? A randomized controlled trial of a linkworker intervention. *J Public Health Med* 1994;16(2):179-85.

For peer review only - http://bmjopen.bmj.com/site/about/guidelines.xhtml

76. Grewal S, Bottorff JL, Balneaves LG. A Pap test screening clinic in a South Asian community of Vancouver, British Columbia: challenges to maintaining utilization. *Public Health Nurs* 2004;21(5):412-8.
77. Lofters AK, Vahabi M, Prakash V, et al. Lay health educators within primary care practices to improve cancer screening uptake for South Asian patients: Challenges in quality improvement. *Patient Preference and Adherence* 2017;11:495-503. doi: http://dx.doi.org/10.2147/PPA.S127147
78. Kinnaird V, Momsen J. Different Places, Different Voices: Gender and Development in Africa, Asia and Latin America. London and New York: Routledge 2002
79. Senarath U, Gunawardena N. Women's Autonomy in Decision Making for Health Care in South Asia. *Asia Pac J Public Health* 2009;21(2):137-43.
80. Malvia S, Bagadi SA, Dubey US, Saxena S. Epidemiology of breast cancer in Indian women. Asia-Pacific J Clin Oncol 2017; 13:289-295.
81. DH. NHS Cancer Screening, 2015
82. Jain A, Serevitch J. Breast cancer screening - how do we communicate with women of South Asian origin? . *Breast Cancer Research* 2009 11(Suppl 2):1. doi: https://doi.org/10.1186/bcr2400

For peer review only

For peer review only - http://bmjopen.bmj.com/site/about/guidelines.xhtml

Table 1: Inductive studies				
**Region**	**Sample size**	**Sampling frame**	**Focus**	**Findings**
**Breast cancer**				
Ahmad et al, (2012)				
Toronto Canada	60 Indian and Pakistani immigrant women, 50+ years; never screened or screened >3 years ago	Concept mapping. Clustering of participant-generated statements.	Experiences and beliefs concerning barriers to mammography	Barriers to regular screening mammogram: lack of knowledge; fear of cancer and language and transportation. Barriers differed significantly according to years lived in Canada: dependence on family; ease of access to mammogram centre; language and transportation; fear of cancer and self-care.
Bottorff et al (1998)				
Large urban setting, Western Canada	50 SA women, 30+ years. FGDs with 30 mostly new informants.	IDIs and FGDs with healthy immigrant SA women via SA investigators' Networks	Beliefs attitudes and values related to breast health practices and screening	Beliefs centred on 4 domains:1) A woman's calling - keeping the family honour, modesty and putting others first; 2) Beliefs about cancer.; 3) Taking care of your breasts; 4) Accessing services.
Meana et al (2001)				
Toronto, Canada	30 recently immigrated Tamil women from Sri Lanka ≥ 50 years	Members of a SA Women's Centre. 3 FGDs	Attitudes/ beliefs regarding BC and BC screening	Common barriers to BC screening: 1) lack of understanding of the role of early detection in medical care; 2) Religious beliefs; 3) Fear of social stigmatisation. Other barriers: embarrassment about mammography procedures. No reported opposition from husbands.

For peer review only - http://bmjopen.bmj.com/site/about/guidelines.xhtml

Pons-Vigués, et al. (2012a)				
Barcelona, Spain	68 healthy women (6 Pakistani, Indian women), 40-69 years	Key informants, cultural mediators and associations.	Concept of health prevention and knowledge, perceived benefits/ barriers	Health prevention concept lay across 3 axes. 1) *Understanding of prevention*; 2) *Proactive or deterministic conception of health disease*: *3)* women cared little for their own health but obliged to others.
**Cervical cancer**				
Bottorff et al. (2001)				
Western Canada	20 SA (Sikh, Hindu, Muslim) women; 20+ years, had Pap test.	IGIs with SA women attending for Pap testing organised by ethnic group	Experiences and views concerning testing, their expectations and preferences	Perceptions of Pap testing: uncertainty about benefits of early detection in the absence of symptoms; reservations about screening unmarried young women due to preserving virginity; seen as beneficial to keep healthy and protect families from disease. Interplay between cultural values and healthcare system structures: shyness and discomfort discussing Pap test with physician .
Haworth et al (2014a)				
Nebraska mid-western USA	27 healthy Bhutanese refugee women; 19 - 60 years.	Snowball sample community venues and residences (2 FGDs)	CC and screening knowledge; susceptibility severity of CC; benefits/ barriers to screening.	Most women had never heard of CC (or HPV) and felt it did not occur in their community. Women not familiar with concept of health prevention. Barriers: shyness; feelings of exposure and potential stigma; historical abuse, sexual assault and inappropriate behaviour by male HCPs in refugee camps; language; navigating a complex health system; limited insurance coverage; transportation; male translators.
Oelke & Vollman (2007)				

For peer review only - http://bmjopen.bmj.com/site/about/guidelines.xhtml

Urban Canada	53 immigrant Sikh women, 21 to 65+ years. Residency in Canada 6 months - 32 years	Community locations, key contacts and Punjabi radio (13 IDIs). Community agency and English classes (3 FGDs)	Knowledge, understanding and perceptions of CC screening	*'Inside/outside'*: difficult to move 'outside' into Canadian society. *Individual*: unaware of importance of prevention; cervix as unknown body part; SRH not discussed. *Knowledge:* minimal knowledge of Pap test and no ready access to information. *Prevention*: not necessary in absence of symptoms. *Family:* cultural constraints; domination by males/elders; needing permission for medical appointments; a woman's sacrifice for the family. *Community:* preserving honour/status; shame surrounding inappropriate topic. *Healthcare system:* sex of physician; language barriers; trust; confidentiality and dearth of acceptable HCPs.
**Breast and Cervical Cancer**				
Hulme et al (2016)				
Canada	20 Bangladeshi women (12 individual interviews, 8 in focus groups), 30-65, Residency in Canada; 7 <5 yrs, 7 ≥5yrs, 6 NA.	Selected from participants at a community-based education programme	Knowledge, perceptions of barriers, role of family physicians and preferences for future access	Risk perception associated with personal experience, screening poorly understood in absence of symptoms;language barriers important; role of family physicians important, particularly females (who administer) cervical screening; fear of cancer inhibits screening; importance of self-efficacy, particularly in how self-efficacy is reflected in personal identity.

KEY for all tables:

SA = South Asian
IDI = In-depth Interviews
FGD = Focus Group Discussions

For peer review only - http://bmjopen.bmj.com/site/about/guidelines.xhtml

HCP = Healthcare provider or professional
SRH = Sexual and reproductive health
BC = Breast Cancer
CC = Cervical Cancer

HA= Health Ambassador

For peer review only

For peer review only - http://bmjopen.bmj.com/site/about/guidelines.xhtml

Table 2 Predictive studies						
**Region**	**Sample Size**	**Sampling Frame**	**Focus**	**Outcome Variable**	**Rate**	**Risk Factors for not screening**
**Breast Cancer Study**						
Ahmed & Stewart, 2004						
Canada	Cross-section 54 SA women, aged 18+, Hindu or Urdu speakers	Attendees family medical clinic	HBM	Ever had CBE	38.5%	Younger age, more barriers
Boxwala, et al. 2010						
Detroit, USA	Cross-section 160 Indian women, 39+ years	Cultural or religious locations	HBM	Mammogram and CBE within 2 years	63.8%	Not graduate education, disagree screening useful, mammogram less relatively important, not recommended by health professional
Chawla, et al. 2015						
California, USA	Cross-section 186 SA women aged	Random digit	None	Mammography	79.5	Not married, <25% of lifetime in USA, no

For peer review only - http://bmjopen.bmj.com/site/about/guidelines.xhtml

	50 to 74	telephone survey		test within 2 years		physician visits in last year
Hasnain, et al. 2014						
Chicago, USA	Cross-section 105 SA first generation Muslim women	Snowball sample	Anderson Behavioural Model for Health Service Use Anderson Model), HBM, Transtheoretical Model	Mammography test within 2 years (adherent), Mammography test not within 2 years (overdue), never screened	41% Adherent,	Fewer years in the USA, lower mammogram importance, more barriers, lower intention
Islam, et al. 2006						
New York, USA	Cross-section 43 women 18+	Attendees at cultural events	None	Mammogram test within 2 years	55.8%	Uninsured, greater than ten years living in USA
Kwok, et al 2015						
Sydney, Australia	Cross-section 242 women 18+ born in India or in Indian communities	Attendees at cultural events	Culture-specific factors	Have biannual mammography	17.8%	Less time in Australia, divorced, separated, widowed
Marfani, et al. 2013						
Baltimore, USA	Cross-section 418	Attendees at cultural and	Acculturation	Mammography or CBE within 1	Not	Low self and outcome efficacy for screening,

For peer review only - http://bmjopen.bmj.com/site/about/guidelines.xhtml

	Indian women	religious events		year, Mammography or CBE within 2 years, mammography or CBE more than 2 years ago	provided	greater barriers, lower acculturation, lower acculturation interacting with greater anxiety about BC
Meana, et al. 2001						
Canada	Cross-section 122 Tamil women, 50+ years	Attendees at community and religious centres	HBM	Had mammograms	57.4%	Fewer years in North America, More barriers
Menon, et al. 2012						
Chicago, USA	Cross-section 330 SA women 40+ years	Community-based agencies	Precede-Proceed Model	Ever had mammography	65.5%	Less than 5 years in USA, greater barriers, lower English language preference, never had cervical screening
Misra, et al. 2011a						
USA cities	Cross-section 389 Indian women 40+	Random survey	None	Ever had mammography	81.2%	Fewer years in USA, No health insurance
Misra, et al. 2011b						

For peer review only - http://bmjopen.bmj.com/site/about/guidelines.xhtml

USA cities	Cross-section 519 Indian women 18+	Random survey	None	Ever had Pap test	74.2%	Fewer years in USA, Lower education, No health insurance, No family cancer history
Pourat, et al., 2010						
California, USA	Cross-section 134 SA women 40+	Random survey	Acculturation	Mammogram within 2 years	39%	None
Vahabi, et al. (2016)						
Ontario, Canada	18,880 women 50-69	Government. Database linkage study	None	Verified mammography attendance within 2 years	63.7%	Fewer years in Canada, no general GP assessment, GP trained overseas
Vahabi, et al. (2017)						
Ontario, Canada	14,352 women 50-74	Government. Database linkage study	Muslim majority country of origin	Verified mammography attendance within 2 years	44.02% Muslim majority country, 45.41% non-Muslim majority	Muslim majority country of origin, male family doctor, family class immigrant, not speaking English and French, fee-for-service primary care or no primary care
**Cervical Screen Study**						

For peer review only - http://bmjopen.bmj.com/site/about/guidelines.xhtml

Chaudhry, et al. 2003						
USA	Cross-section 225 SA women aged 15-83	SA Family Names	Anderson Model	Pap test within 3 years	73%	Unmarried, not bachelor degree, no usual source of medical care, <25% of lifetime in USA
Chawla, et al. 2015						
California, USA	Cross-section 711 SA women aged 21 to 74	Random digit telephone survey	None	Pap test within 3 years	79.5	Younger age, not married, <25% of lifetime in USA
Gupta, et al., 2002						
Toronto, Canada	Cross-section 62 SA university students, 62 Tamil women 18-60	Common areas of university, Tamil community centres	Acculturation	Ever had Pap test	25%	Lower education, education outside Canada, lower acculturation
Islam, et al. 2006						
New York, USA	Cross-section 98 women 18+	Attendees at cultural events	None	Pap test within 3 years	54.4%	Tested within 3 years: Lower education, Lower income, Uninsured, less than ten years living in USA
Kue, Hanegan & Tan (2017)						

For peer review only - http://bmjopen.bmj.com/site/about/guidelines.xhtml

Columbus Ohio, USA	Cross-section 97 Bhutanese-Nepali refugees18+	Convenience sample at community locations	Beliefs, barriers and post-migration difficulties.	Ever had Pap test	44.3%	Not positive perceptions of test, greater barriers, not recommended by HCP family or friends, fewer post-migration difficulties
Lin, et al., 2009						
California, USA	Cross-section 338 SA women 18-65	Random telephone survey	None	Pap test in last 3 years	73%	Not married, low income, no usual source of medical care
Lofters et al (2017)						
Canada		Government. Database linkage study	Muslim majority country of origin	Verified Pap test in last 3 years		Muslim majority country of origin, lowest income male family doctor. Family doctor not Canadian graduate, family class immigrant, not speaking French, fee-for-service primary care or no primary care
Marlow et al. (2017)						
UK	Cross-section of 230 SA women	Cluster randomized community survey	Precaution Adoption Process Model	Four group classification; unaware,	79%	

For peer review only - http://bmjopen.bmj.com/site/about/guidelines.xhtml

		of UK addresses		unengaged, undecided, intention to be screened.		
Menon, et al. 2012						
Chicago, USA	Cross-section 330 SA women 40+ years	Community-based agencies	Precede-Proceed Model	Ever had cervical screen	32.8%	Lower education, greater barriers, lower English language preference, never had mammogram
Misra, et al. 2011						
USA cities	Cross-section 519 Indian women 18+	Random survey	None	Ever had Pap test	74.2%	Fewer years in USA, Lower education, No health insurance, No family cancer history
Pourat, et al., 2010						
California, USA	Cross-section 195 SA women 40+	Random survey	Acculturation	Pap test within 3 years	73%	Greater distance to Asian clinic, No health insurance, No private doctor, Has previously delayed obtaining medical care, Has had problem obtaining satisfactory doctor over past year

For peer review only - http://bmjopen.bmj.com/site/about/guidelines.xhtml

Table 3: Comparative studies				
**Region**	**Sample size**	**Sampling frame**	**Focus**	**Findings**
**Breast cancer**				
Abdul Hadi, et al. (2010)				
Penang State, Malaysia	65 healthy Indian women aged >15, 177 Malay and 121 Chinese	Two shopping malls	Differences in knowledge/perception of breast cancer	Indians less knowledge risk factors, symptoms and screening options (subsidised mammography and CBE) compared to Malay and Chinese. Univariate analysis confounded by Indian population being least educated.
Pons-Vigués, et al. (2012b)				
Barcelona city, Spain	25 Pakistani-Indian women 45-69 years, 275 Spanish women 660 other immigrant groups	Sampled from Census respondents.	Adapted HBM based on qualitative pilot study (Pons-Vigues, et al, 2012a).	Indian-Pakistani women perceived more barriers to mammography screening than host country women, but fewer than other immigrant groups.
Sim et al. (2009)				
Singapore	80 Indian women, 182 Malay, 700 Chinese, 38 other	Visitors to general hospital (not patients)	Knowledge and beliefs about BCa and screening practices	No differences between Indian women and others in either knowledge or having ever attended a screening mammogram.
Teo et al. (2013)				
Singapore	52 locally raised Indian women	Female patients and visitors to polyclinic,	No theoretical model	Indian women less likely to have ever had mammogram compared with majority

For peer review only - http://bmjopen.bmj.com/site/about/guidelines.xhtml

	104 Chinese<br>52 Malay	aged 40-70.		Chinese, but more likely than Malays. Indian women *least* likely group to cite cost or potential pain as barriers to attending mammography
Vahabi, et al. (2016)				
Ontario, Canada	18,880 South Asian, 85,872 other immigrant groups	Government database linkage study	No theoretical model	Lower mammography attendance in previous 2 years than other immigrant groups
Wu et al. (2006a)				
Michigan, USA	38 Indian women aged ≥40<br>X Chinese<br>X Filipino	Community or religious groups; ethnic student associations, community events	HBM.	No difference in CBE and mammography take up between ethnic groups. Indian women had lower scores on perceived susceptibility and seriousness than Filipino and Chinese controlling income. Indian women more likely to say 'do not know where to find mammogram'.
Wu et al. (2008b)				
Michigan, USA	109 Asian Indians aged ≥40 years, literate	Community events, cultural centres, faith-based organizations, Asian health fairs	HBM.	No group differences.
**Cervical cancer**				
Dunn & Tan (2010)				

For peer review only - http://bmjopen.bmj.com/site/about/guidelines.xhtml

Malaysia	96 married Indian women aged 25-65	2-stage stratified-cluster random sampling	No theoretical model	Ever had Pap test: Indian population least likely to have ever had screening. Indian women who had ever received screening less likely to know its purpose than Malays. Indian women who had never had Pap test were 9% less likely to cite 'embarrassed' as reason for not undergoing testing
Marlow, Wardle and Waller (2015)				
England, UK	120 Indian, 120 Pakistani, 120 Bangladeshi women, 120 white British, 120 Caribbean and 120 African	Quota sampling, random sampling within high ethnic concentration postcodes	No theoretical model	Indian, Pakistani and Bangladeshi women less likely to be screened over last 5 years than white British. Less knowledge than white British
So et al (2017)				
Hong Kong	161 Indian, Napali and Pakistani women, 959 Chinese women, 50+	Community centres or associations, Chinese sample recruited using random digit dialling	No theoretical model	Sa women less likely to have been screened, had fewer tests in previous 6 years, longer time since last test

For peer review only

For peer review only - http://bmjopen.bmj.com/site/about/guidelines.xhtml

Table 4: Intervention studies				
**Region**	**Sample Size and Sampling frame**	**Intervention**	**Focus**	**Findings**
**Breast cancer**				
Ahmad et al, 2005				
Toronto, Canada	127 SA immigrant women. Mean age = 37 years (SD 9.7); lived 6 years in Canada (SD 6.6). n=82 pre-intervention; n=74 post intervention	Pre (PrI)- Post (PoI) intervention comprising written socioculturally tailored language-specific health education materials.	Barriers to mammography screening<br><br>HBM ,Stages of Change model)	A significant increase in self-reporting 'ever had' routine physical check-up (46.4-70.8%; p < 0.01) and CBE (33.3-59.7%; p < 0.001). Decrease in: misperception of low susceptibility to breast cancer women (3.0-2.4; p < 0.001); misperception of short survival after diagnosis (2.7-1.8; p < 0.001); and perceived barriers to CBE (2.5-2.1; p < 0.001). Self-efficacy to have CBE increased (3.1-3.6; p < 0.001).
Hoare et al, 1994				
Oldham, UK	5277 women with SA names from general practices with high number of SA patients. Pakistani/intervention n=145 (59%); Bangladeshi/controls n=87 (57%).	RCT: 527 women stratified into Pakistani (n=324) intervention and Bangladeshi (n=203) control groups	Awareness of screening	No difference in attendance was found between the intervention and control groups (49%and 47%). Attendance for screening was related to length of stay in the United Kingdom.
Sadler et al, 2003				

For peer review only - http://bmjopen.bmj.com/site/about/guidelines.xhtml

San Diego County, USA	Asian and Pacific Islander women from San Diego County. Indian n=125. Women aged >20 years (screening from 20 onwards)	Pre and post intervention The Asian Grocery Store based cancer intervention program - incorporating an educational program into women's routine shopping activities	Barriers to mammography screening	Shift toward screening uptake for Chinese and Vietnamese American women who were non-adherent at baseline but no change for Asian Indian and Japanese American women at follow up.
**Cervical Cancer**				
Grewal et al, 2004				
Vancouver Canada	Specialised Pap Test clinic for SA women. 1995-1998; 61 – 107 – 35 new visits in the intervention. Reasons for non-attendance n=74.	Time series of service use. Community initiative led by SA community health nurses in collaboration with influential women in the SA community, local physicians, and health board authorities. Qualitative interviews with 20 women who attended the Pap test clinic	Awareness of screening	Attendance patterns were not maintained although women had positive experiences. Challenges for ongoing success: 1) maintaining the continued involvement of stakeholders in developing long-term strategies to enhance community awareness about cervical cancer; 2) creating mechanisms to strengthen support from physicians in the community; 3) meeting the needs of the underserved within a specialised health service for SA immigrant women.
McAvoy & Raza, 1991				

For peer review only - http://bmjopen.bmj.com/site/about/guidelines.xhtml

Leicester, UK	737 randomly selected Asian women; 18-52 years who were not recorded on the central cytology computer as ever having had a cervical smear. N=578 (declined n=159)	Prospective Cohort RCT study (blinded trial)<br>1) visited, shown a video, n=263;<br>2) visited, shown a leaflet and fact sheet, n=219;<br>3) posted a leaflet and fact sheet, n=131;<br>4) not contacted at all, n=124.	Knowledge of early intervention	Only 6 (5%) of those not contacted and 14 (11%) of those sent leaflets had a smear test during the study. Health education interventions increased the uptake of cervical cytology among women in Leicester who had never been tested. Visits and videos were most effective.
Ornelas et al, 2017				
Greater Seattle US	40 SA women, 20 Karen-Burmese and 20 Nepali-Bhutanese; 21-58 years (mean age = 35years); living in US for 5 years on average. Most did not speak English well or at all (75%); 8 years average of education; 65% married.73% had Pap test since arriving to US, 70% in last 3 years.	Pre and post survey<br>The two health educators recruited participants through personal contacts they had in their community, as well as referrals from community advisors and participants with whom they had completed data collection. A pilot study to evaluate the acceptability and efficacy of the 17 minute videos provided in their native language.	Behavioural Model<br>Changes in cervical cancer awareness, intention to be screened for cervical cancer, cervical cancer-related knowledge	Nepali-Bhutanese were significantly more likely to have been screened than Karen-Burmese (90% v 55%). Women showed significant increases in knowledge for all the individual items, as well as the mean composite knowledge scores (5.6 to 9.3, $p < .001$) after viewing the video. There were also increased in knowledge for individual items across ethnic groups; however, not all were significant. Mean changes in the knowledge score were significant for women in each ethnic group (5.4 to 9.2, $p < .001$ for Karen-Burmese and 5.8 to 9.5, $p < .001$ for Nepali-Bhutanese). Women indicated high satisfaction with the video length and very few women reported about anything they did not like.

For peer review only - http://bmjopen.bmj.com/site/about/guidelines.xhtml

**Breast and cervical cancer**				
Kernohan 1996				
Bradford UK	October 1991 to March 1993, a stratified sample of 1,000 women (670 SA, 163 African-Caribbean, 96 Eastern European and 71 other)	Community development approach - Pre - Post intervention<br>Two Health Promotion Facilitators undertook community development work in both formal and informal settings. Women were interviewed at the beginning of the project and six months after the health promotion intervention.	Knowledge about cervical cancer and breast cancer	SA women had the lowest levels of knowledge and also showed the most significant improvements. Significant increases in attendance for cervical smear and breast cancer screening were self-reported.
Lofters et al, 2017				
Ontario Canada	624 phone calls made; of which 257 were to SA women. 129 (50%) of SA women spoken to directly by SA HAs.	3 quality improvement initiatives for 4 Physicians using a snowballing technique:<br>1) Educational vidoes shown in the waiting room and/or 1-1 education with patients by SA HAs<br>2) 1-1 education with	Transtheoretical Model<br>Barriers and facilitators to cervical and mammography screening	Most SA women spoken to by a SA HA indicated a willingness to get screened for breast or cervical cancer and some went on to action their screening intention. Making phone calls to patients to invite them for screening had the most reach and most appeal. The initiatives were reported to be resource intensive for physicians even with voluntary SA HAs involved. However, using SA HAs showed promise to increase awareness and willingness

For peer review only - http://bmjopen.bmj.com/site/about/guidelines.xhtml

		patients identified by SA HAs or Physician<br>3) Phone calls to patients by SA HAs		to be screened for cancer.

For peer review only

For peer review only - http://bmjopen.bmj.com/site/about/guidelines.xhtml

Figure 1: PRISMA Flowchart of the study selection process. Adapted from: PLoS Medicine (OPEN ACCESS) Moher D, Liberati A, Tetzlaff J, Altman DG, The PRISMA Group (2009). Preferred Reporting Items for Systematic Reviews and Meta-Analyses: The PRISMA Statement. PLoS Med 6(7): e1000097. doi:10.1371/journal.pmed1000097.

For peer review only

For peer review only - http://bmjopen.bmj.com/site/about/guidelines.xhtml

For peer review only

For peer review only - http://bmjopen.bmj.com/site/about/guidelines.xhtml

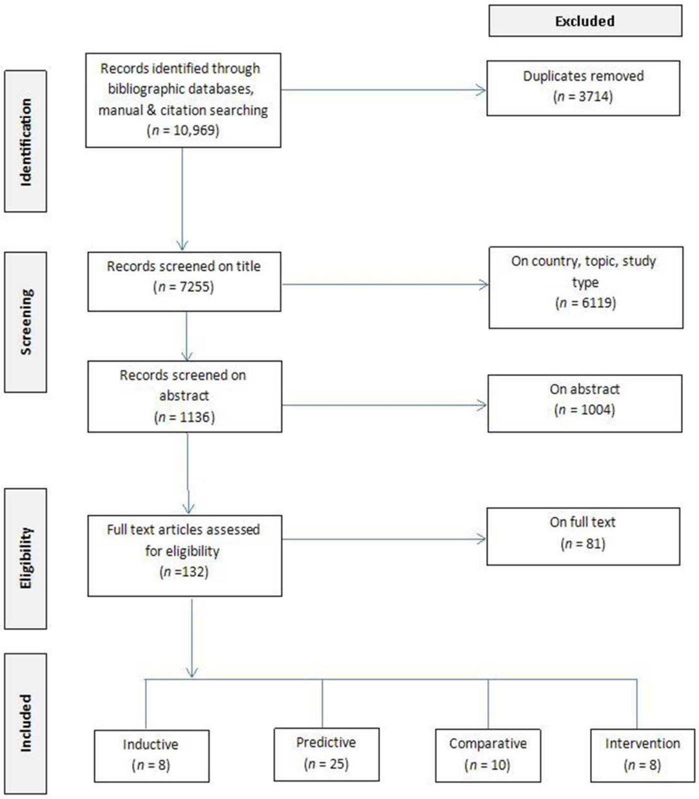

201x284mm (100 x 100 DPI)

PRISMA 2009 Checklist

Section/topic	#	Checklist item	Reported on page #
**TITLE**			
Title	1	Identify the report as a systematic review, meta-analysis, or both.	1
**ABSTRACT**			
Structured summary	2	Provide a structured summary including, as applicable: background; objectives; data sources; study eligibility criteria, participants, and interventions; study appraisal and synthesis methods; results; limitations; conclusions and implications of key findings; systematic review registration number.	2-3
**INTRODUCTION**			
Rationale	3	Describe the rationale for the review in the context of what is already known.	5-6
Objectives	4	Provide an explicit statement of questions being addressed with reference to participants, interventions, comparisons, outcomes, and study design (PICOS).	7
**METHODS**			
Protocol and registration	5	Indicate if a review protocol exists, if and where it can be accessed (e.g., Web address), and, if available, provide registration information including registration number.	3, 8
Eligibility criteria	6	Specify study characteristics (e.g., PICOS, length of follow-up) and report characteristics (e.g., years considered, language, publication status) used as criteria for eligibility, giving rationale.	8-9
Information sources	7	Describe all information sources (e.g., databases with dates of coverage, contact with study authors to identify additional studies) in the search and date last searched.	8
Search	8	Present full electronic search strategy for at least one database, including any limits used, such that it could be repeated.	8
Study selection	9	State the process for selecting studies (i.e., screening, eligibility, included in systematic review, and, if applicable, included in the meta-analysis).	8-9
Data collection process	10	Describe method of data extraction from reports (e.g., piloted forms, independently, in duplicate) and any processes for obtaining and confirming data from investigators.	9-11
Data items	11	List and define all variables for which data were sought (e.g., PICOS, funding sources) and any assumptions and simplifications made.	10
Risk of bias in individual studies	12	Describe methods used for assessing risk of bias of individual studies (including specification of whether this was done at the study or outcome level), and how this information is to be used in any data synthesis.	10
Summary measures	13	State the principal summary measures (e.g., risk ratio, difference in means).	10
Synthesis of results	14	Describe the methods of handling data and combining results of studies, if done, including measures of consistency (e.g., I^2) for each meta-analysis.	11

For peer review only - http://bmjopen.bmj.com/site/about/guidelines.xhtml

PRISMA 2009 Checklist

Section/topic	#	Checklist item	Reported on page #
Risk of bias across studies	15	Specify any assessment of risk of bias that may affect the cumulative evidence (e.g., publication bias, selective reporting within studies).	10
Additional analyses	16	Describe methods of additional analyses (e.g., sensitivity or subgroup analyses, meta-regression), if done, indicating which were pre-specified.	11
**RESULTS**			
Study selection	17	Give numbers of studies screened, assessed for eligibility, and included in the review, with reasons for exclusions at each stage, ideally with a flow diagram.	12
Study characteristics	18	For each study, present characteristics for which data were extracted (e.g., study size, PICOS, follow-up period) and provide the citations.	31-49
Risk of bias within studies	19	Present data on risk of bias of each study and, if available, any outcome level assessment (see item 12).	na
Results of individual studies	20	For all outcomes considered (benefits or harms), present, for each study: (a) simple summary data for each intervention group (b) effect estimates and confidence intervals, ideally with a forest plot.	31-49
Synthesis of results	21	Present results of each meta-analysis done, including confidence intervals and measures of consistency.	na
Risk of bias across studies	22	Present results of any assessment of risk of bias across studies (see Item 15).	na
Additional analysis	23	Give results of additional analyses, if done (e.g., sensitivity or subgroup analyses, meta-regression [see Item 16]).	12-19
**DISCUSSION**			
Summary of evidence	24	Summarize the main findings including the strength of evidence for each main outcome; consider their relevance to key groups (e.g., healthcare providers, users, and policy makers).	20-24
Limitations	25	Discuss limitations at study and outcome level (e.g., risk of bias), and at review-level (e.g., incomplete retrieval of identified research, reporting bias).	21
Conclusions	26	Provide a general interpretation of the results in the context of other evidence, and implications for future research.	22-24
**FUNDING**			
Funding	27	Describe sources of funding for the systematic review and other support (e.g., supply of data); role of funders for the systematic review.	25

From: Moher D, Liberati A, Tetzlaff J, Altman DG, The PRISMA Group (2009). Preferred Reporting Items for Systematic Reviews and Meta-Analyses: The PRISMA Statement. PLoS Med 6(7): e1000097. doi:10.1371/journal.pmed1000097

For more information, visit: www.prisma-statement.org.

For peer review only - http://bmjopen.bmj.com/site/about/guidelines.xhtml
